# Effectiveness of behaviour change techniques in lifestyle interventions for non-communicable diseases: an umbrella review

**DOI:** 10.1186/s12889-024-20612-8

**Published:** 2024-11-07

**Authors:** Iliatha Papachristou Nadal, Chaisiri Angkurawaranon, Ankur Singh, Yanee Choksomngam, Vidhi Sadana, Loren Kock, Apichai Wattanapisit, Nutchar Wiwatkunupakarn, Sanjay Kinra

**Affiliations:** 1 Non-Communicable Disease Epidemiology, London School of Hygiene & Tropical Medicine, Keppel Street, London, WC1E 7HT UK; 2 Division of Long-Term Conditions, King’s College London, James Clerk Maxwell Building, 57 Waterloo Rd, London, SE1 8WA UK; 3 Department of Family Medicine, Faculty of Medicine, Chiang Mai University, 110 Intavaroros, Sriphum, Muang District, Chiang Mai, 50200 Thailand; 4 Global Health and Chronic Conditions Research Group, Chiang Mai University, 239, Huay Kaew Road, Muang District, Chiang Mai, 50200 Thailand; 5 Faculty of Population Health Science, University College London, 1-19 Torrington Place, London, UK; 6 School of Medicine, Walailak University, 222 Thaiburi, Thasala District Nakhon Si, Thammarat, 80160 Thailand

**Keywords:** Behaviour change techniques, Lifestyle interventions, Non-communicable diseases, Umbrella review

## Abstract

**Objective:**

To identify the most commonly reviewed behaviour change techniques (BCTs) and their effectiveness based on consistency across reviews for lifestyle interventions of non-communicable diseases.

**Design:**

Umbrella review of systematic reviews.

**Data sources:**

PubMed, Embase, PsycINFO, Cochrane CENTRAL, Global Health.

**Data extraction and synthesis:**

A narrative synthesis of extracted findings was conducted. The Behaviour Change Technique v1 Taxonomy was used to identify and code behaviour change techniques (e.g., goal setting) in a standardised manner, which were independently assessed by two reviewers. Study quality was independently assessed by two reviewers using the assessment of multiple systematic review tools.

**Results:**

26 reviews were included with a total of 72 BCT labels evaluated across the different lifestyle interventions and non-communicable diseases. A total of 13 BCT clusters were identified to be reported as effective. The most commonly reviewed BCTs and their effectiveness/ineffectiveness were as follows: ‘Goals and Planning’ (12 effective/1 ineffective), ‘Feedback and monitoring’ (9 effective/3 ineffective), ‘Social support’ (9 effective/1 ineffective), ‘Shaping knowledge’ (11 effective/1 ineffective), and ‘Natural consequences’ (6 effectiveness/ 2 ineffective). The vast majority of the studies were conducted in high-income and a few in upper middle-income countries, with hardly any studies from lower middle-income and lower income studies.

**Conclusion:**

The most common BCTs were ‘Goals and Planning’, ‘Feedback and Monitoring’, ‘Shaping Knowledge’, ‘Social Support’, and ‘Natural Consequence’. Based on consistency across reviews, several BCTs such as ‘Goals and Planning’, Feedback and Monitoring’, ‘Shaping Knowledge’, and ‘Social Support’ have demonstrated effectiveness (Recommendation Grade A) in improving health behaviours across a limited range of NCDs. The evidence is less clear for other BCT techniques. It is also likely that not all BCTs will be transferable across different settings. There is a need for more research in this area, especially in low-middle-income countries.

**Protocol registration:**

Registered on the International Prospective Register of Systematic Reviews; PROSPERO (CRD42020222832).

**Supplementary Information:**

The online version contains supplementary material available at 10.1186/s12889-024-20612-8.

## Backgound

Non-communicable diseases (NCDs), such as diabetes and cardiovascular disease, are estimated to account for 73% of global deaths, with this burden expected to grow over the coming decade [1, 2]. The surging death rates from NCDs are considerably higher in low- and middle-income countries (LMICs) than in high-income countries, with more than three-quarters of global NCD deaths (32 million) occurring in LMICs [2]. Although mortality attributed to NCDs is found in older age groups in high-income countries, 85% of the 15 million deaths attributed to NCDs occur between 30 years and 69 years in LMICs [3]. Much of this morbidity and mortality is attributed to unhealthy lifestyle behaviours such as tobacco smoking, physical inactivity, poor diet and medication non-adherence, along with related metabolic risks (high blood pressure, obesity, high cholesterol, and blood sugar) [1]. However, changing to healthier behaviours and maintaining them is difficult. In this context, lifestyle interventions can play an important role towards reducing the burden of NCDs globally [4–6]. Lifestyle interventions can be complex, often comprising multiple interacting components [7, 8] and have shown effectiveness in preventing unhealthy lifestyle behaviours and promoting healthy protective behaviours [9].

The Medical Research Council (MRC) guidelines recommend including behavioural change theory within complex intervention study designs [10]. This is because previous research has shown that studies that are theoretically based are more effective in increasing participant adherence to treatment advice compared to those that are not theoretically based [11–13]. However, despite the MRC’s guidelines, there is still a scarcity of theory-driven studies in healthcare [14]. One such use of theory-informed behaviour change intervention design involves the behaviour change technique (BCT) taxonomy [15]. The BCT v1 taxonomy (93 BCT labels grouped into 16 clusters) helps researchers identify the active ingredients for effective lifestyle interventions [15, 16].

Different types of BCTs are usually required for different lifestyle interventions. Reviews have shown that the most effective lifestyle interventions that include self-management techniques improve behaviours such as improving diet, increasing physical activity and reducing smoking and/or excessive alcohol consumption [17, 18].’In terms of smoking cessation, techniques promoting commitment, social reward and identity were associated with changed behaviour [19]. However, to prevent postpartum smoking relapse, techniques that addressed problem solving and how to maintain abstinent behaviour, health information provision, and social support were found to be most effective [20]. Additionally, in diabetes prevention programmes with high levels of retention found focusing on changing behaviour as a more useful technique than focusing on the outcome such as weight [21]. However, self-monitoring was not found to be effective to improve retention or reduce diabetes incidence [21].

While a number of systematic reviews have examined the effectiveness of BCT on lifestyle interventions, BCT classification may differ across reviews. The aim of this umbrella review is to identify (a) which BCTs are most commonly assessed in systematic reviews and (b) what is the evidence of their effectiveness based on consistency of the findings across these reviews, with a particular focus on NCDs.

## Methods

The umbrella review methodology was applied to synthesise evidence from multiple systematic reviews creating a single ‘meta review’ with results from the included systematic reviews being used as the raw data [22]. The benefit of an umbrella review versus a systematic review is that data can be compared across different interventions and conditions, providing a broader summary of the findings [23]. In addition, umbrella reviews can assess reasons for conflicting results across reviews.

The protocol was registered on PROSPERO (CRD42020222832).

## Eligibility criteria

The studies were selected and included based on the following inclusion and exclusion criteria:

### Inclusion criteria

#### Study characteristics

Systematic reviews and meta-analyses published since 1946 to date of search (October, 2023) were included. Eligible systematic reviews primarily included randomized controlled trials (RCTs), however primary studies of any experimental or quasi-experimental study design were also included providing the authors had identified studies using systematic methods. Publications reporting on populations from both high income as well as low-middle-income countries were included. Eligibility was limited to reviews available in English.

## Subject/Participants

Reviews with adult patients (18 years or older) with one or more of the main non-communicable diseases as defined by the World Health Organisation (2018) [3] were eligible. These include cardiovascular diseases (such as coronary heart disease and stroke), cancers, chronic respiratory diseases (such as chronic obstructive pulmonary disease and asthma), diabetes, chronic kidney diseases, chronic musculoskeletal conditions (such as arthritis, back pain and problems, osteoporosis) [3].

## Interventions

Reviews with lifestyle interventions, defined as any intervention that included: physical activity; healthy eating/diet; smoking cessation; reducing excessive drinking; adherence to treatment, including medication and attending medical appointments were eligible [24, 25]. These interventions included behavioural change techniques and/or behavioural change theory, with the primary outcome to improve or maintain clinical or behaviour changes. Interventions could be implemented in any health or social care setting (primary, secondary or community care). The community settings encompassed care given in the community, in patient homes or by health and social care professionals.

## Exclusion criteria

Studies were excluded if they involved children and/or adolescents, participants who had a mental health condition (including dementia), specified mental health as a primary outcome (e.g., reducing depression and anxiety), if the lifestyle interventions did not include was not defined, interventions that addressed infectious diseases, studies where interventions did not include a behaviour change techniques or behaviour change theory.

### Outcome measures

Studies assessing effect on at least one of the primary/secondary outcomes listed below as change from baseline and difference between intervention and control groups were included:

1. Primary: NCD-related clinical or subclinical parameters/biomarkers such as disease signs and symptoms, weight, body mass index (BMI), blood pressure, blood lipids (total cholesterol, lipoprotein, triglycerides), blood glucose levels (fasting/post prandial), glycated haemoglobin (HbA1c).
2. Secondary: behavioural risk factors such as physical activity levels, dietary intake, smoking cessation, or adherence to intervention.

## Search strategy

A comprehensive search was conducted, with the most appropriate search terms for specific database namely, PubMed; Embase, PsychINFO, Global Health, and Cochrane CENTRAL (Supplementary file 1)). The databases were chosen to ensure that all relevant literature was identified. In addition, the reviewers checked references of the included systematic reviews for additional relevant studies. Finally, the following fields were integrated into the search strategy: Language, study type, publication date, and full-text availability.

Searches were performed in October 2020 and repeated in February 2021 and October 2023.

## Eligibility assessment and data extraction

The results from all literature searches were imported into Mendeley reference management software. From all retrieved indexed records, duplicate records were eliminated by the software and then unique records were imported into a web-based systematic review software package,

Covidence (Veritas Health Innovation). Firstly, two authors (IPN and AS) independently screened titles and abstracts against the inclusion and exclusion criteria. Second, all the studies that met the inclusion criteria were reviewed through a full-text screening process by the 2 authors independently. Disagreements were resolved through discussions or where necessary by an independent reviewer (CA). Additional studies were identified for inclusion by reviewing the reference lists of the included systematic reviews. A further relevance check of all the screened articles was done. Data were extracted into Excel using a pre-defined data collection form by two reviewers (IPN and AS) from included studies. The form was first piloted on a sample of studies external to the review to ensure consistency of extraction between authors.

Using the form, data on review characteristics (year of the study, countries, healthcare settings and NCD(s) focused on), aims and objectives (primary and secondary), methodology (study design, number of studies included, inclusion and exclusion criteria, search strategy, database searched, number and type of participants and their disease condition), intervention characteristics (intervention type, frequency and types of different BCTs and psychological theories/models employed, intervention and follow-up duration and delivery mode/technique), outcomes of interest (primary and secondary), generalizability, scalability, limitations and implications of the study were extracted from each included review and crosschecked by IPN and AS. Discrepancies between reviewers’ extracted data were resolved via discussion, or by a third reviewer (CA) if necessary.

### Coding of BCTs

The BCTs were coded into the 93 BCT labels and then grouped into 16 distinct sets of BCTs, known as clusters [26]. These clusters were grouped based on similarity of their active ingredient and are as follows (with number of BCT labels in parentheses): scheduled consequences (10), reward/threat (7), repetition/replacement (7), antecedents (4), associations (8), covert learning (3), natural consequences (6), feedback and monitoring (5), goals and planning (9), social support (3), comparison of behaviour (3), self-belief (4), comparison of outcomes (3), identity (5), shaping knowledge (4) and adjunctive (4).

Studies reporting that they did not follow the BCT v1 taxonomy were recoded according to the BCT v1 taxonomy [27], using the definitions of BCTs labels and clusters. BCT coding was performed independently by two reviewers. Any questions about the coding were resolved by discussion and consensus between the reviewers. The reviewers coded all BCTs reported across the reviews. The BCTs were reported as BCT labels and those BCT that were reported as effective were coded into clusters. In addition, the reviews that reported BCTs as ineffective or unknown effectiveness were coded into clusters.

### Quality appraisal of systematic reviews

The methodological quality of the included systematic review was performed using the assessment of multiple systematic reviews (AMSTAR 2) tool [28]. The study quality was categorised based on the overall AMSTAR 2 rating of high, moderate and low The quality for each review was independently assessed by IPN and VS.

### Data analysis

#### Data items

The following general information was extracted and tabulated from the included articles: author name(s), publishing journal, year of publication, country/region, type of studies in included systematic reviews, study aims/objectives/questions, the setting, study population, inclusion and exclusion criteria, type of intervention, behaviour change techniques included, psychological theories/models in use, intervention characteristics like delivery mode/technique, duration of intervention and follow-up, delivery personnel, primary and secondary outcomes, findings, limitations, generalizability and implications of the study.

### Data synthesis

The data was summarised and descriptively analysed for key characteristics of the populations, lifestyle interventions and most commonly reviews behaviour change techniques, their effectiveness/ineffective based on consistency across reviews and key outcomes reported in the included publications. The effectiveness of the BCTs used was graded based on the level of evidence of practice recommendations: Grade A = strong recommendation, Grade B = recommendation, C = option, and D = option. Grade A implies that the evidence was supported by systematic reviews of high-quality randomized controlled trials (RCT), prospective cohort studies or by multiple consistent findings from systematic reviews of lesser quality RCTs, prospective cohorts, retrospective cohorts, untreated controls from RCTs, case-control studies, and case series. Grade B was defined as generally consistent findings from systematic reviews of lesser quality RCTs, prospective cohorts, retrospective cohorts, untreated controls from RCTs, case-control studies, and case series. Grade C involves inconsistent findings from systematic reviews of lesser quality prospective cohorts, retrospective cohorts, untreated controls from RCTs, case-control studies, and case series. Lastly, Grade D refers to little or no systematic empirical evidence [29].

No quantitative syntheses (e.g., meta-analyses) of intervention effectiveness were undertaken due to the heterogeneity of available data. We narratively synthesised information on commonly reviewed behaviour change techniques and their use in interventions for the management of NCDs, classifying them according to the disease category, and countries along with the effectiveness. We narratively synthesised information on behavioural change techniques according to their use by different types of lifestyle interventions (such as physical activity; healthy eating/diet; smoking cessation) and according to disease category. The reporting of the manuscript follows the guideline as suggested by the Preferred Reporting Items for Systematic Reviews and Meta-analyses (PRISMA) statement [30].

### Patient and public involvement

Patients and/or the public were not involved in the design, or conduct, or reporting or dissemination plans of this research.

## Results

**Fig. 1:**
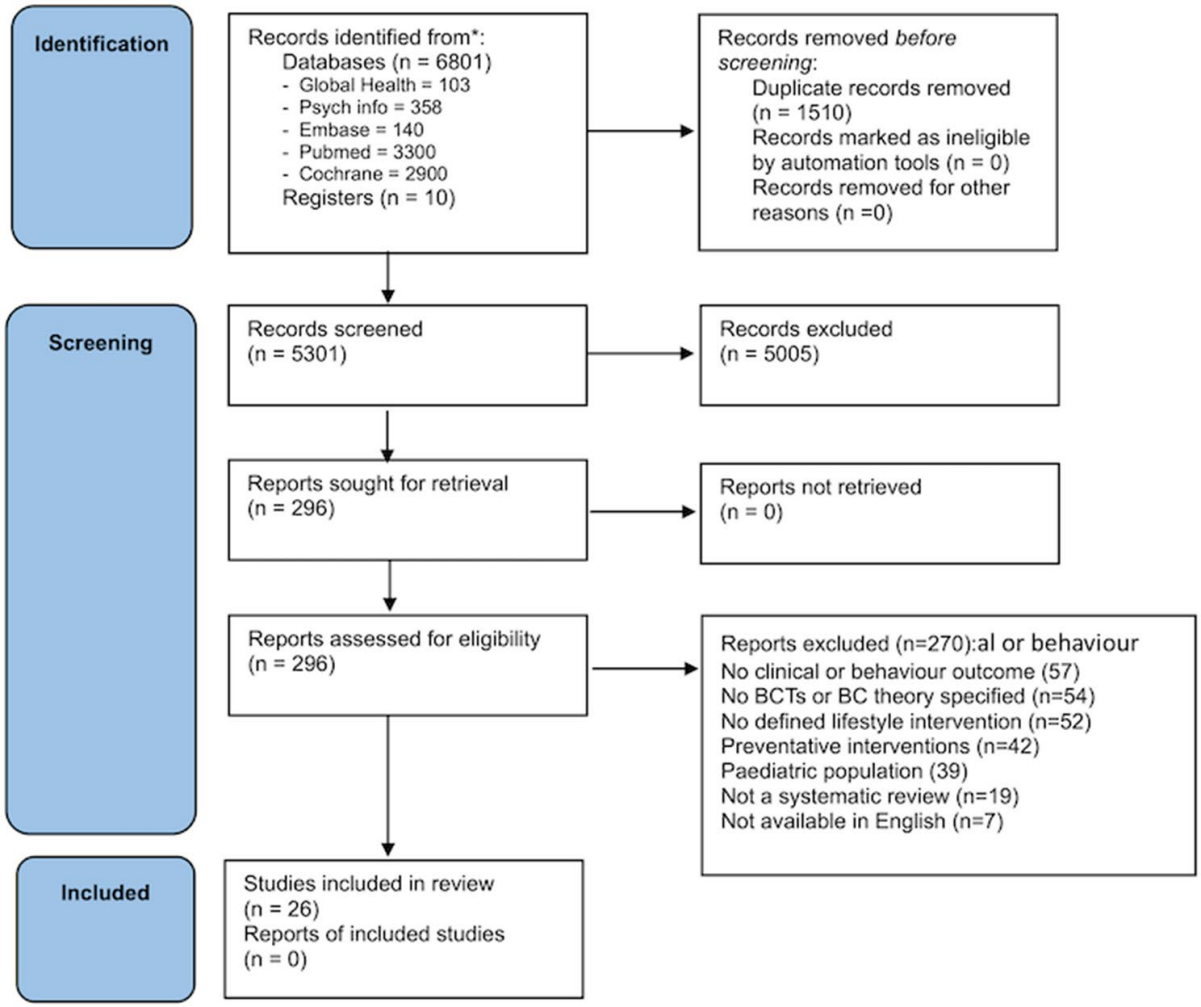
PRISMA 2020 flow diagram for new systematic reviews which included searches of databases and registers only

We identified a total of 6,801 reviews. Following the removal of duplicates, 5,301 reviews were screened based on their titles and abstracts by two authors. Subsequently, 296 reviews underwent a full-text screening. Disagreement during abstract and full-text screening was resolved by a third senior author. Ultimately, 26 systematic reviews were included in the analysis. The PRISMA flowchart of the search is displayed as Fig. 1.

A total of 26 systematic reviews encompassed 614 original articles with diverse study designs, including longitudinal, feasibility, mixed-method, non-control; controlled clinical trials, quasi-experimental design, pre-experimental designs, pre- and post-designs, cluster-RCTs, and quasi-RCTs. All systematic reviews incorporated randomized controlled trials (RCTs), with 18 reviews exclusively driven by RCTs. Additionally, eleven meta-analyses were conducted (Supplementary file 2).

All 26 systematic reviews were published between 2004 and 2020. The majority of the primary studies included in the systematic reviews were carried out in high-income countries, with a focus on the USA, Australia, and the UK. A limited number of studies were conducted in upper-middle-income countries such as Algeria, Brazil, India, Iran, Malaysia, Turkey, South Africa, and China. However, few studies conducted in lower-middle-income countries, India, and very few were conducted in low-income countries. Follow-up periods for the intervention varied widely, ranging from 30-minute single follow-up time to multiple follow-up time over 10 years. Additionally, many did not specify follow-up duration (Additional file 2).

The most studied condition was diabetes (*n* = 9) followed by cancer (*n* = 5); respiratory conditions (*n* = 3) including chronic obstructive pulmonary disease (COPD), asthma and sleep apnoea; cardiovascular diseases (CVD) (*n* = 3), including hypertension; persistent musculoskeletal pain/chronic musculoskeletal (MSK) (*n* = 4) including fibromyalgia and rheumatoid arthritis; obesity with co-morbidities (*n* = 1) and chronic kidney (*n* = 1) (Additional file 2).

## Quality of included reviews

The results of the methodological quality assessment according to the AMSTAR 2 tool are presented in Supplementary file 3. From the 26 reviews six reviews were rated high, 17 medium and three low. The AMSTAR 2 items that most of the included references failed to meet were the following: the report of the review did not contain an explicit statement that the review methods were established prior to the conduct of the review and did not report any significant deviations from the protocol; review authors did not provide a list of excluded studies nor justify the exclusions; and review authors did not report on the sources of funding for the studies included in the review. The majority of the reviews are rated high to moderate in terms of design quality. These are demonstrated from the inclusion of PICO components for the research question and inclusion criteria; a comprehensive literature search strategy; description of included studies in adequate detail as well as risk of bias in individual studies.

### Use of behaviour change techniques and theories

Of the 26 included reviews, 25 reported BCTs, one reported behaviour change theories only [31], and four reported BCTs and behaviour change theories. Nineteen of the reviews that reported BCTs applied the BCT v1 taxonomy, one review used the smoking cessation BCT model by Michie, Churchill, et al. (2011) [32], and six reviews did not follow the BCT v1 taxonomy. Rather than reporting BCTs, these six reviews used counselling frameworks i.e., cognitive behavioural theory (CBT), motivational interviewing and other techniques such as mindfulness, and relaxation. All but one review [33] reported BCTs and their effectiveness and/or ineffectiveness. The most used behaviour change theory across all twenty-five reviews studied social cognitive theory. Other theories that were most frequently used were the Transtheoretical model, Chronic care model and Control theory. Other models and approaches used were the COPE model; e-Health belief model; Pender model of health promotion and self-efficacy. All theories and models are reported in Supplementary file 2).

### Most commonly reported BCTs and their effectiveness/ineffectiveness

The most reviewed BCTs clusters were ‘Goals and Planning’ (*N* = 15), ‘Feedback and Monitoring’ (*N* = 12), ‘Shaping Knowledge’ (*N* = 12), ‘Social Support’ (*N* = 10), and ‘Natural Consequences’ (*N* = 9). Other BCTs found in our reviews were ‘Repetition and Substitution’ (*N* = 5), ‘Comparison of Behavior’ (*N* = 3), ‘Comparison of Outcomes’ (*N* = 3), ‘Associations’ (*N* = 3), ‘Regulation’ (*N* = 2), ‘Self-belief’ (*N* = 2), ‘Antecedents’ (N = 1), and ‘Identity’ (N = 1) respectively.

### Effectiveness of BCTs in lifestyle interventions

In this review, we categorized the lifestyle interventions into five types: treatment adherence, physical activity, smoking cessation, combined diet and physical activity, and combined diet, physical activity, smoking cessation. The description of lifestyle intervention was summarized in Table 1.

**Table 1:** Lifestyle intervention groupings

Intervention group	Description of intervention
Physical activity (PA) (*n* = 10)	Home based cardiac rehab programme using education manuals, e-health and HCP delivery; PA delivered through e-health only; PA combined with structured counselling; Structured exercises using a range of modes such as walks, telephone coaching, books, group and self-management programme; Aerobic training, resistance training and yoga; PA education combined psychoeducational, with mobile phone-based peer support; supervised practical sessions, consisted of aerobic, strengthening and range of motion exercises, both land and water based; aerobic relaxation and home exercise programmes; exercise with motivation enhancement and counselling
Adherence to treatment and/or medication (*n* = 9)	Online coaching programs to self-monitor blood glucose levels, including professional feedback and advice; self-management educational, tele-monitoring combined with counselling; self-care management, information, decision-making skills and counselling and support; behavioural modification to manage glycaemic control, combined with counselling such as CBT, relaxation techniques and stress management; diabetes telephone and face to face coaching to improve glycaemic control; asthma self-care consultation, including stress management for reducing unscheduled health care use; medication adherence and measuring blood pressure; managing glucose control through counselling such as CBT, relaxation therapy;
Combined lifestyle interventions (*n* = 6) (Diet and physical activity combined; *n* = 5). (Diet, physical activity, and smoking cessation; *n* = 1)	PA moderate intensity, resistant training, and dietary counselling through various mode of delivery; PA moderate intensity, strength training, reduction of calories; educational goal setting and planning; nutritional change in dietary habits, dietary regime, physical activity habits physiotherapy and weight training exercise combined with counselling; weight monitoring, calorie intake, PA self-monitoring and social support; Dietary counselling and coaching e.g. water intake, sodium restrictions, aerobic and resistance training, care by a multi-disciplinary team using a telehealth device, group and one to one home visits, including smoking cessation advice;
Smoking cessation (*n* = 1)	Smoking cessation using both advice and medication for people with COPD.

The effectiveness of BCTs across different interventions was presented in the levels of evidence (Grade A-D), illustrated in Table 2. The summary of effectiveness for common BCTs are summarized below. The details of effective BCTs for different diseases is included as Supplementary file 4–7.

**Table 2:**
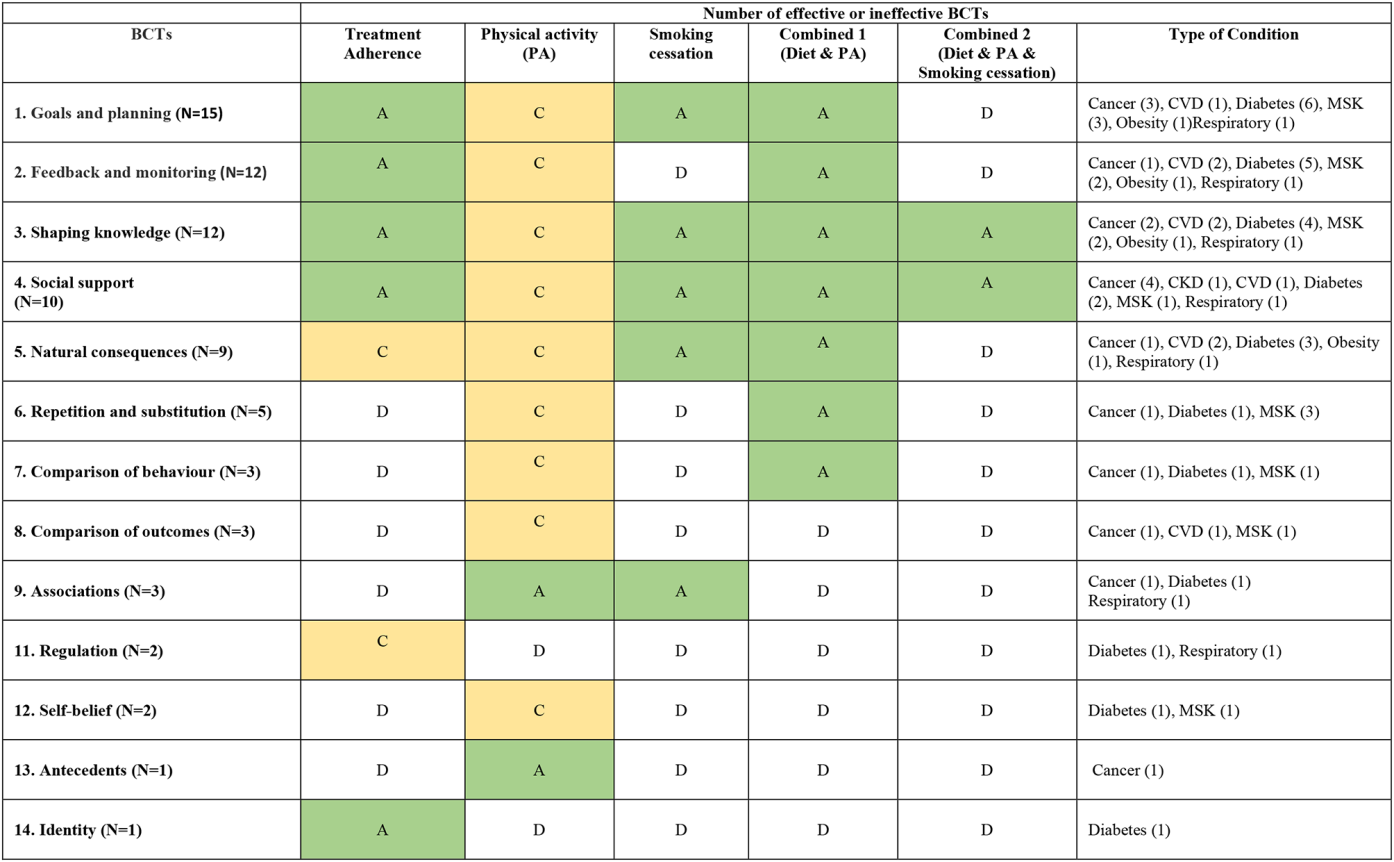
A summary of all the most common reviewed BCTs by level of evidence

Level of effectiveness: Grade A - the evidence was supported by systematic reviews of high-quality RCTs, prospective cohort study or by *multiple consistent findings* from multiple systematic reviews of lesser quality RCTs, prospective cohort, retrospective cohort, untreated controls from RCT, case-control study, and case series: Grade B: *generally consistent findings* from systematic reviews of lesser quality RCTs, prospective cohort, retrospective cohort, untreated controls from RCT, case-control study, and case series. Grade C: *inconsistent findings* from systematic reviews of lesser quality RCTs, prospective cohort, retrospective cohort, untreated controls from RCT, case-control study, and case series. Ultimately, Grade D refers to little or no systematic empirical evidence

Abbreviations: CVD-cardiovascular disease, MSK – musculoskeletal diseases

### Goals and planning

Fifteen of the 19 reviews reported ‘Goals and planning’ and was the most commonly reviewed BCT. There is strong evidence (Recommendation grade A) to support that ‘goals and planning’ are effective for interventions focusing on treatment adherence [34–37]., smoking cessation [38] and combined diet and physical activity [39–42]. The support of the use of this technique in interventions focusing on physical activity (Recommendation grade C) [43–48] or more complex interventions targeting multiple aspects of lifestyle interventions (Recommendation grade D).

### Feedback and monitoring

Twelve reviews mentioned the use of feedback and monitoring techniques. Evidence supports that ‘Feedback and monitoring’ can be an effective BCT (Recommendation grade A) for improving treatment adherence among patients with diabetes and respiratory diseases [35–37, 49–51]. There is also strong evidence (Recommendation grade A) to support ‘Feedback and monitoring’ as an effective BCT for improving patient eating habits and physical activity [41, 42]. The evidence to support ‘Feedback and monitoring’ as an effective BCT for interventions targeting physical activity alone is less clear (Recommendation Grade C). While some studies showed increasing exercise in patients with cardiovascular disease (CVD), some reported non-significant improvement [52].

### Shaping knowledge

This technique was reported in 12 reviews. Evidence supports its effectiveness (Recommendation Grade A) for promoting treatment adherence [49], smoking cessation [38], and multiple lifestyle modifications (e.g., diet control, physical activity, smoking cessation, and quality of life improvement). Using ‘Shaping knowledge’ as a BCT technique in counselling programs have effectively improved patient outcomes for those with cancer, diabetes mellitus, obesity, and chronic kidney disease [42, 54, 55]. Its effectiveness in enhancing physical activity is still inconclusive (Recommendation grade C) [43, 52, 53].

### Social support

Ten reviews focused on social support techniques. It was found that using ‘Social support’ as a BCT has been successful in improving treatment adherence in patients with diabetes mellitus [34] (Recommendation grade A), in smoking cessation in patients with respiratory conditions [38] (Recommendation grade A), and in a combination of diet, physical activity, and smoking cessation interventions among patients with cancer and chronic kidney diseases [41, 54, 55] (Recommendation grade A). However, there was inconsistent evidence to support its use for improving physical activity alone [43–46, 53] (Recommendation grade C).

### Natural consequences

Ten reviews reported the use of the natural consequence method. Evidence supports this technique was effective (Recommendation grade A) for smoking cessation in patients with respiratory conditions [38] and for combined diet and physical activity interventions in patients with cancer [54]and obesity [42]. However, its benefits in improving patient treatment adherence [34, 36, 56] and physical activity [43–45] remained unclear (Recommendation grade C).

### Other BCTs

Five or fewer reviews out of 26 reported the effectiveness of the following BCTs: repetition and substitution (5); comparison of behaviour (2); comparison of outcomes (3); associations (3); regulation (2); antecedents (1); self-belief (1); identity (2).

#### Repetition and substitution

There was mixed evidence on the use of ‘Repetition and substitution’ as an effective BCT in targeting physical activity alone [44, 46, 47, 57]. (Recommendation grade C) while stronger evidence supports its use in interventions targeting both diet and physical activity [40] (Recommendation grade A).

#### Comparison of behaviour

Two review provided evidence that **‘**Comparison of behaviours’ is an effective BCT for interventions targeting physical activity and diet [40, 53] (Recommendation grade A). The evidence for the use of this BCT for intervention physical activity alone is less clear [57]. (Recommendation grade C)

### Comparison of outcomes

Three reviews examine the effectiveness of ‘Comparison of outcomes’ as a BCT for interventions targeting physical activity. There was inconsistent evidence across different conditions as it was found to be effective among patients with diabetes and cardiovascular disease [52, 53] but less clear for patients with musculoskeletal disorders [57]. (Recommendation grade C).

### Association

Two reviews provided evidence that using ‘Association’ as a BCT was effective for interventions targeting physical activity among patients with diabetes [37] and cancer [53] (Recommendation grade A). Another review focused on respiratory conditions and reported ‘Association’ as an effective BCT for smoking cessation [38]. (Recommendation grade A).

#### Regulation

Two reviews focused on treatment adherence of which one review reported regulation as an effective BCT among patients with diabetes [50]. The evidence was less clear in the second review examining the use of ‘Regulation” as a BCT for interventions targeting adherence to medication among patients with respiratory conditions [51]. (Recommendation grade C).

#### Self-belief

Two reviews focused on physical activity. One of the two reviews assessed patients with diabetes and reported ‘Self-belief’ as an effective BCT for increasing physical activity [43]. However, the evidence provided by a second review that assessed patients with musculoskeletal disorders was less clear [48]. (Recommendation grade C).

#### Antecedents and identity

One review reported ‘Antecedents’ as an effective BCT in physical activity interventions among patients with cancer [53] (Recommendation grade A). One review reported ‘Identity’ as effective an effective BCT for treatment adherence among patients with diabetes [50] (Recommendation grade A).

## Discussion

The primary objective of this study was to identify the most commonly reviewed BCTs and their effectiveness based on consistency across reviews for lifestyle interventions aiming to reduce NCDs. The most commonly reported BCT clusters were: ‘Goals and Planning’, ‘Feedback and monitoring’, ‘Social support’, ‘Shaping knowledge’, and ‘Natural consequences’. The vast majority of the studies were conducted in high-income countries, and a few were conducted in upper middle-income countries, with few studies from lower middle-income and lower income countries.

Overall, this umbrella review is supportive of the Spring et al. meta review where goal setting and feedback and monitoring were the most effective BCTs for improving lifestyle outcomes [24]. The consistency of the findings from this review supports previous research reporting BCTs that increases effectiveness of interventions compared to those do not report BCTs [4, 58, 59].

Furthermore, we have identified core BCTs used for targeted lifestyle changes (e.g., increasing physical activity within CVD or improving diet within diabetes). thereby gaining a better understanding of the causal mechanisms of an intervention needed to achieve effective clinical and behavioural outcomes [60].

### Strengths and weaknesses

The findings of the review should be considered in the context of certain strengths and limitations. This umbrella review was registered with and guided by PROSPERO and was conducted according to PRISMA guidelines. Furthermore, a considerable strength of this umbrella review was an attempt to search and identify all relevant systematic reviews by adopting a well-framed and designed comprehensive search strategy, using multiple databases. The authors also hand-searched review papers and references to identify additional studies that may have relevance to this review. This umbrella review therefore endeavours to collate evidence that embraces and envelops all relevant existing systematic reviews (therefore all included primary studies) with a focus on lifestyle interventions in secondary and tertiary management of a wide range of NCDs. It attempts to analyse, consolidate, and summarise the usefulness and caveats in the application of BCTs and theories used in lifestyle interventions to reduce disease progression and improve outcomes. It also summarises the most effective BCTs, derived from a careful and systematic analysis of systematic reviews.

In terms of its limitation, firstly, as an umbrella review, the study was restricted to the analyses reported in the included reviews and did not re-examine or re-analyse primary studies. Thus, the gaps identified in the evidence base for some BCT interventions may reflect the lack of systematic reviews of these strategies rather than the absence of primary studies. Another potential limitation of this umbrella review is that it does not describe the current state of research sufficiently, since there might have been single randomised controlled studies published that are yet to be included into systematic review articles.

The inclusion criteria are restricted to interventions targeting secondary and tertiary management of diseases. While BCT-based interventions targeting primary prevention of diseases are essential and could have yielded us more studies, their incorporation could have reduced the rigour of the current review. This is because the use of BCTs for secondary and tertiary management of diseases may use difference strategies and interventions to that of primary prevention management interventions.

Another key area is addressing the issue relating to the poor and insufficient description of behaviour change interventions and its inconsistent use of terminology. There is substantial variation in terminology used for several behaviour change techniques. This variation in terminology used makes the coding of the techniques used difficult when reviewing behaviour change interventions. We have used literature available and made significant efforts to convert differently named BCTs into Behaviour Change Technique Taxonomy version 1 (BCTTv1) by Michie et al. 2013 [61] This has enabled us to bring all BCTs on the same plane for their hierarchical coding and comparative analysis. Therefore, there is a need to have consistent terminology and sufficient information on intervention components to allow for the replication of lifestyle interventions that have been found to be effective.

## Gaps in research

The included systematic reviews largely examined behaviour change interventions that were more commonly implemented in high-income countries, and most of the primary studies included in these systematic reviews were from high-income countries, reflecting the available published literature. Hence, the replicability and application of the findings with regards to certain behaviour change techniques and interventions may be limited and cannot be generalised globally.

## Conclusion

The primary objective of this study was to identify the most commonly reviewed BCTs and their effectiveness. The most common BCTs found in this study were ‘Goals and Planning’, ‘Feedback and Monitoring’, ‘Shaping Knowledge’, ‘Social Support’, and ‘Natural Consequence’. Based on consistency across reviews, several BCTs such as ‘Goals and Planning’, Feedback and Monitoring’, ‘Shaping Knowledge’, and ‘Social Support’ have demonstrated effectiveness (Recommendation grade A) in improving health behaviours across a limited range of NCDs. The evidence is less clear for other BCT techniques. It is also likely that not all BCTs will be transferable across different settings. There is a need for more research in this area, especially in low-middle-income countries.

## Electronic supplementary material

Below is the link to the electronic supplementary material.

Supplementary Material 1

Supplementary Material 2

Supplementary Material 3

Supplementary Material 4

Supplementary Material 5

Supplementary Material 6

Supplementary Material 7

## Data Availability

All data generated and analysed are available in the supplementary files.

## Abbreviations

BCTs: Behaviour change techniques
NCDs: non-communicable diseases
AMSTAR: assessment of multiple systematic reviews
LMICs: low-income and middle-income countries
MRC: Medical Research Council
RCTs: randomized controlled trials
BMI: body mass index
HbA1c: glycated haemoglobin
CBT: cognitive behavioural theory
COPD: chronic obstructive pulmonary disease
CVD: cardiovascular diseases
QA: quality assessment
MSK: musculoskeletal
PROSPERO: International Prospective Register of Systematic Reviews
PRISMA: Preferred Reporting Items for Systematic Reviews flowchart of the searches

## References

[1] Coates MM et al. (2020) ‘Burden of non-communicable diseases from infectious causes in 2017: a modelling study’. 10.1016/S2214-109X(20)30358-2 10.1016/S2214-109X(20)30358-2 PMC8040338 33098769

[2] GBD 2017 Causes of Death Collaborators, Bernabe E, Adekanmbi V. Global, regional, and national age-sex-specific mortality for 282 causes of death in 195 countries and territories, 1980–2017: a systematic analysis for the global burden of Disease Study 2017. Lancet. 2018;392(10159):1736–88. 10.1016/S0140-6736(18)32203-7. 30496103 10.1016/S0140-6736(18)32203-7 PMC6227606

[3] World Health organisation. (2018) *Noncommunicable diseases*. https://www.who.int/news-room/fact-sheets/detail/noncommunicable-diseases (Accessed: 8 April 2021).

[4] Jepson RG, et al. The effectiveness of interventions to change six health behaviours: a review of reviews. BMC Public Health BioMed Cent. 2010;10(1):538–538. 10.1186/1471-2458-10-538. 10.1186/1471-2458-10-538 PMC2944371 20825660

[5] Fjeldsoe B, et al. Systematic Review of Maintenance of Behavior Change following physical activity and dietary interventions’, *Health psychology*. Am Psychol Association. 2011;30(1):99–109. 10.1037/a0021974. 10.1037/a0021974 21299298

[6] Samdal GB. (2019) *Behaviour change interventions in primary health care*. dissertation. The University of Bergen.

[7] Davidson K, et al. Evidence-based behavioral medicine: what is it and how do we achieve it? Annals of behavioral medicine. Volume 26. New York: Springer-; 2003. pp. 161–71. 310.1207/S15324796ABM2603_01. 10.1207/S15324796ABM2603_01 14644692

[8] Craig P, et al. Developing and evaluating complex interventions: the new Medical Research Council guidance’, *International journal of nursing studies*. Elsevier Ltd. 2020;50(5):587–92. 10.1016/j.ijnurstu.2012.09.010. 10.1016/j.ijnurstu.2012.09.010 23159157

[9] Michie S, et al. Evaluating the effectiveness of behavior change techniques in health-related behavior: a scoping review of methods used. Translational behavioral medicine. Volume 8. Oxford University Press; 2018. pp. 212–24. 210.1093/tbm/ibx019. 10.1093/tbm/ibx019 PMC6062857 29381786

[10] Skivington K, Matthews L, Simpson SA, Craig P, Baird J, Blazeby JM, et al. A new framework for developing and evaluating complex interventions: update of Medical Research Council guidance. BMJ. 2021;374(n2061). 10.1136/bmj.n2061. 10.1136/bmj.n2061 PMC8482308 34593508

[11] French SD, et al. Evaluation of a theory-informed implementation intervention for the management of Acute Low Back Pain in General Medical Practice: the IMPLEMENT Cluster Randomised Trial. PLoS ONE. 2013;8(6). 10.1371/journal.pone.0065471. 10.1371/journal.pone.0065471 PMC3681882 23785427

[12] French DP, et al. Which Behaviour Change techniques are most effective at increasing older adults’ self-efficacy and physical activity Behaviour? A systematic review. Annals of behavioral medicine. Volume LLC. Springer Science and Business Media; 2014. pp. 225–34. 210.1007/s12160-014-9593-z. 10.1007/s12160-014-9593-z 24648017

[13] Dziedzic KS et al. (2014) *Implementing the NICE osteoarthritis guidelines: a mixed methods study and cluster randomised trial of a model osteoarthritis consultation in primary care - the Management of OsteoArthritis In Consultations (MOSAICS) study protocol*. 10.1186/s13012-014-0095-y 10.1186/s13012-014-0095-y PMC4176866 25209897

[14] Painter J, et al. The Use of Theory in Health Behavior Research from 2000 to 2005: a systematic review. Annals of behavioral medicine. Volume 35. New York: Springer-; 2008. pp. 358–62. 310.1007/s12160-008-9042-y. 10.1007/s12160-008-9042-y 18633685

[15] Michie S, et al. The behavior change technique taxonomy (v1) of 93 hierarchically clustered techniques: building an International Consensus for the reporting of Behavior Change interventions. Annals of behavioral medicine. Volume 46. Boston: Springer US; 2013. pp. 81–95. 110.1007/s12160-013-9486-6. 10.1007/s12160-013-9486-6 23512568

[16] Davis R et al. (2014) ‘Theories of behaviour and behaviour change across the social and behavioural sciences: a scoping review’, *Health psychology review*. Routledge, 9(3), pp. 323–344. 10.1080/17437199.2014.941722 10.1080/17437199.2014.941722 PMC4566873 25104107

[17] Michie S et al. (2009) ‘Effective techniques in healthy eating and physical activity interventions: a Meta-regression’, 28(6), pp. 690–701. 10.1037/a0016136 10.1037/a0016136 19916637

[18] Michie S et al. (2010) ‘Development of a taxonomy of behaviour change techniques used in individual behavioural support for smoking cessation’. 10.1016/j.addbeh.2010.11.016 10.1016/j.addbeh.2010.11.016 21215528

[19] Black N, et al. Behaviour change techniques associated with smoking cessation in intervention and comparator groups of randomized controlled trials: a systematic review and meta-regression’, *addiction*. Blackwell Publishing Ltd. 2020;115(11):2008–20. 10.1111/add.15056. 10.1111/add.15056 32196796

[20] Brown TJ, et al. A systematic review of behaviour change techniques within interventions to prevent return to smoking postpartum. Addictive behaviors. Elsevier Ltd; 2019. pp. 236–43. 10.1016/j.addbeh.2018.12.031. 10.1016/j.addbeh.2018.12.031 PMC6518963 30731328

[21] Begum S, Povey R, Ellis N, Gidlow C. A systematic review of recruitment strategies and behaviour change techniques in group-based diabetes prevention programmes focusing on uptake and retention. Diabetes Res Clin Pract. 2020;166:108273. 10.1016/j.diabres.2020.108273. Epub 2020 Jun 23. PMID: 32590009. 32590009 10.1016/j.diabres.2020.108273

[22] Damery S, Flanagan S, Combes G. Does integrated care reduce hospital activity for patients with chronic diseases? An umbrella review of systematic reviews. BMJ Open. 2016;6(11). 10.1136/bmjopen-2016-011952. 10.1136/bmjopen-2016-011952 PMC5129137 27872113

[23] Pieper D, Antoine SL, Mathes T, Neugebauer EAM, Eikermann M. Systematic review finds overlapping reviews were not mentioned in every other overview. J Clin Epidemiol. 2014;67(4):368–75. 10.1016/j.jclinepi.2013.11.007. 24581293 10.1016/j.jclinepi.2013.11.007

[24] Spring B, Moller AC, Coons MJ. Multiple health behaviours: overview and implications. J Public Health. 2012;34:I3–10. 10.1093/pubmed/fdr111. 10.1093/pubmed/fdr111 PMC3284863 22363028

[25] Martin S, Hagger, Kyra Hamilton. Effects of socio-structural variables in the theory of planned behavior: a mediation model in multiple samples and behaviors. Psychol Health. 2020;0:0. 10.1080/08870446.2020.1784420 32608265

[26] Michie S et al. Behaviour change techniques: the development and evaluation of a taxonomic method for reporting and describing behaviour change interventions (a suite of five studies involving consensus methods, randomised controlled trials and analysis of qualitative Da. Heal Technol Assess 19, (2015). 10.3310/hta19990 PMC4781650 26616119

[27] Abraham C, Michie S. A taxonomy of Behavior Change techniques used in interventions. Health Psychol. 2008;27(3):379–87. 10.1037/0278-6133.27.3.379. 18624603 10.1037/0278-6133.27.3.379

[28] Shea BJ, Reeves BC, Wells G, Thuku M, Hamel C, Moran J, Moher D, Tugwell P, Welch V, Kristjansson E, Henry DA. AMSTAR 2: a critical appraisal tool for systematic reviews that include randomised or non-randomised studies of healthcare interventions, or both. BMJ. 2017;358:j4008. 10.1136/bmj.j4008. PMID: 28935701; PMCID: PMC5833365. 28935701 10.1136/bmj.j4008 PMC5833365

[29] Burns PB, Rohrich RJ, Chung KC. The levels of evidence and their role in evidence-based medicine. Plast Reconstr Surg. 2011;128(1):305–10. 10.1097/PRS.0b013e318219c171. 21701348 10.1097/PRS.0b013e318219c171 PMC3124652

[30] Page P, MJ, McKenzie JE, Bossuyt PM, Boutron I, Hoffmann TC, Mulrow CD, et al. The PRISMA 2020 statement: an updated guideline for reporting systematic reviews. BMJ. 2021;372(n71). 10.1136/bmj.n71. 10.1136/bmj.n71 PMC8005924 33782057

[31] McCullough AR, Ryan C, Macindoe C, Yii N, Bradley JM, O’Neill B, Elborn JS, Hughes CM. Behavior change theory, content and delivery of interventions to enhance adherence in chronic respiratory disease: a systematic review. Respir Med. 2016;116:78–84. Epub 2016 May 24. PMID: 27296825. 27296825 10.1016/j.rmed.2016.05.021

[32] Michie S, van Stralen MM, West R. The behaviour change wheel: a new method for characterising and designing behaviour change interventions. Implement Sci BioMed Cent. 2011;6(1):42. 10.1186/1748-5908-6-42. 10.1186/1748-5908-6-42 PMC3096582 21513547

[33] Igwesi-Chidobe CN, Kengne AP, Sorinola IO, Godfrey EL. Physical activity containing behavioural interventions for adults living with modifiable chronic non-communicable diseases in Africa: a systematic mixed-studies review. Int Health. 2018;10(3):137–148. 10.1093/inthealth/ihy013. PMID: 29554307. 10.1093/inthealth/ihy013 29554307

[34] van Vugt M, de Wit M, Cleijne WH, Snoek FJ. Use of behavioral change techniques in web-based self-management programs for type 2 diabetes patients: systematic review. J Med Internet Res. 2013;15(12):e279. 10.2196/jmir.2800. PMID: 24334230; PMCID: PMC3869055. 24334230 10.2196/jmir.2800 PMC3869055

[35] Ismail K, Winkley K, Rabe-Hesketh S. Systematic review and meta-analysis of randomised controlled trials of psychological interventions to improve glycaemic control in patients with type 2 diabetes. Lancet. 2004;363(9421):1589-97. 10.1016/S0140-6736(04)16202-8. PMID: 15145632. 10.1016/S0140-6736(04)16202-8 15145632

[36] Sherifali D, Viscardi V, Bai JW, Ali RM. Evaluating the Effect of a Diabetes Health Coach in Individuals with Type 2 Diabetes. Can J Diabetes. 2016;40(1):84–94. 10.1016/j.jcjd.2015.10.006. PMID: 26827684. 10.1016/j.jcjd.2015.10.006 26827684

[37] Lee YH, Chiou PY, Chang PH, Hayter M. A systematic review of the effectiveness of problem-solving approaches towards symptom management in cancer care. J Clin Nurs. 2011;20(1–2):73–85. Epub 2010 Nov 2. PMID: 21044188. 21044188 10.1111/j.1365-2702.2010.03401.x

[38] Bartlett YK, Sheeran P, Hawley MS. Effective behaviour change techniques in smoking cessation interventions for people with chronic obstructive pulmonary disease: a meta analysis. Br J Health Psychol. 2014;19(1):181–203. 10.1111/bjhp.12071. Epub 2013 Oct 30. PMID: 24397814; PMCID: PMC4253323. 24397814 10.1111/bjhp.12071 PMC4253323

[39] Fredrix M, McSharry J, Flannery C, Dinneen S, Byrne M. Goal-setting in diabetes self-management: a systematic review and meta-analysis examining content and effectiveness of goal-setting interventions. Psychol Health. 2018;33(8):955–77. Epub 2018 Mar 2. PMID: 29498547. 29498547 10.1080/08870446.2018.1432760

[40] Cradock KA, ÓLaighin G, Finucane FM, et al. Behaviour change techniques targeting both diet and physical activity in type 2 diabetes: a systematic review and meta-analysis. Int J Behav Nutr Phys Act. 2017;14:18. 10.1186/s12966-016-0436-0. 28178985 10.1186/s12966-016-0436-0 PMC5299734

[41] Stacey FG, James EL, Chapman K, Courneya KS, Lubans DR. A systematic review and meta-analysis of social cognitive theory-based physical activity and/or nutrition behavior change interventions for cancer survivors. J Cancer Surviv. 2015;9(2):305–38. 10.1007/s11764-014-0413-z. Epub 2014 Nov 29. PMID: 25432633; PMCID: PMC4441740. 25432633 10.1007/s11764-014-0413-z PMC4441740

[42] Dombrowski SU, Falko F, Sniehotta A, Avenell M, Johnston. Graeme MacLennan & Vera Araújo-Soares (2012) identifying active ingredients in complex behavioural interventions for obese adults with obesity-related co-morbidities or additional risk factors for co-morbidities: a systematic review. Health Psychol Rev, 6:1, 7–32, 10.1080/17437199.2010.513298

[43] Avery L, Flynn D, van Wersch A, Sniehotta FF, Trenell MI. Changing physical activity behavior in type 2 diabetes: a systematic review and meta-analysis of behavioral interventions. Diabetes Care. 2012;35(12):2681–9. 10.2337/dc11-2452. PMID: 23173137; PMCID: PMC3507564. 23173137 10.2337/dc11-2452 PMC3507564

[44] Grimmett C, Corbett T, Brunet J, et al. Systematic review and meta-analysis of maintenance of physical activity behaviour change in cancer survivors. Int J Behav Nutr Phys Act. 2019;16:37. 10.1186/s12966-019-0787-4. 31029140 10.1186/s12966-019-0787-4 PMC6486962

[45] Duff OM, Walsh DM, Furlong BA, O’Connor NE, Moran KA, Woods CB. Behavior change techniques in physical activity eHealth interventions for people with Cardiovascular Disease: systematic review. J Med Internet Res. 2017;19(8):e281. 10.2196/jmir.7782. PMID: 28768610; PMCID: PMC5559649. 28768610 10.2196/jmir.7782 PMC5559649

[46] Meade L, Bearne L, Sweeney L, Alageel S, Godfrey E. Behaviour change techniques associated with adherence to prescribed exercise in patients with persistent musculoskeletal pain: systematic review. Br J Health Psychol. 2018;24. 10.1111/bjhp.12324. 10.1111/bjhp.12324 PMC6585717 29911311

[47] Eisele A, Schagg D, Krämer LV, Bengel J, Göhner W. Behaviour change techniques applied in interventions to enhance physical activity adherence in patients with chronic musculoskeletal conditions: a systematic review and meta-analysis. Patient Educ Couns. 2019;102(1):25–36. Epub 2018 Sep 29. PMID: 30279029. 30279029 10.1016/j.pec.2018.09.018

[48] Larkin L, Gallagher S, Cramp F, Brand C, Fraser A, Kennedy N. Behaviour change interventions to promote physical activity in rheumatoid arthritis: a systematic review. Rheumatol Int. 2015;35(10):1631–40. 10.1007/s00296-015-3292-3. Epub 2015 May 21. PMID: 25994094. 25994094 10.1007/s00296-015-3292-3

[49] Winkley K, Upsher R, Stahl D, Pollard D, Brennan A, Heller SR, Ismail K. Psychological interventions to improve glycemic control in adults with type 2 diabetes: a systematic review and meta-analysis. BMJ Open Diabetes Res Care. 2020;8(1):e001150. 10.1136/bmjdrc-2019-001150. PMID: 32273289; PMCID: PMC7254106. 32273289 10.1136/bmjdrc-2019-001150 PMC7254106

[50] Yang X, Li Z, Sun J. Effects of cognitive behavioral therapy-based intervention on improving glycaemic, psychological, and physiological outcomes in adult patients with diabetes Mellitus: a Meta-analysis of Randomized controlled trials. Front Psychiatry. 2020;11:711. 10.3389/fpsyt.2020.00711. PMID: 32848906; PMCID: PMC7399630. 32848906 10.3389/fpsyt.2020.00711 PMC7399630

[51] Denford S, Taylor RS, Campbell JL, Greaves CJ. Effective behavior change techniques in asthma self-care interventions: systematic review and meta-regression. Health Psychol. 2014;33(7):577–87. 10.1037/a0033080. Epub 2013 Jul 1. PMID: 23815765. 23815765 10.1037/a0033080

[52] Heron N, Kee F, Donnelly M, Cardwell C, Tully MA, Cupples ME. Behaviour change techniques in home-based cardiac rehabilitation: a systematic review. Br J Gen Pract. 2016;66(651):e747–57. 10.3399/bjgp16X686617. Epub 2016 Aug 1. PMID: 27481858; PMCID: PMC5033311. 27481858 10.3399/bjgp16X686617 PMC5033311

[53] Hallward L, Patel N, Duncan LR. Behaviour change techniques in physical activity interventions for men with prostate cancer: A systematic review. J Health Psychol. 2020;25(1):105–122. doi: 10.1177/1359105318756501. Epub 2018 Feb 15. PMID: 29446325.Spring, B., Champion, K. E., Acabchuk, R., & Hennessy, E. A. (2020). 10.1177/1359105318756501 29446325

[54] D’Egidio V, Sestili C, Mancino M, Sciarra I, Cocchiara R, Backhaus I, Mannocci A, De Luca A, Frusone F, Monti M, La Torre G, RETURN TO BREAST Collaborative group. Counseling interventions delivered in women with breast cancer to improve health-related quality of life: a systematic review. Qual Life Res. 2017;26(10):2573–92. 10.1007/s11136-017-1613-6. Epub 2017 Jun 16. PMID: 28623442. 28623442 10.1007/s11136-017-1613-6

[55] Evangelidis N, Craig J, Bauman A, Manera K, Saglimbene V, Jaure, Allison. Lifestyle behaviour change for preventing the progression of chronic kidney disease: a systematic review. BMJ Open. 2019;9:e031625. 10.1136/bmjopen-2019-031625. 31662393 10.1136/bmjopen-2019-031625 PMC6830616

[56] Etminani K, Tao Engström A, Göransson C, Sant’Anna A, Nowaczyk S. How Behavior Change strategies are used to design Digital interventions to improve medication adherence and blood pressure among patients with hypertension: systematic review. J Med Internet Res. 2020;22(4):e17201. 10.2196/17201. PMID: 32271148; PMCID: PMC7180506. 32271148 10.2196/17201 PMC7180506

[57] O’Dwyer T, Maguire S, Mockler D, Durcan L, Wilson F. Behaviour change interventions targeting physical activity in adults with fibromyalgia: a systematic review. Rheumatol Int. 2019;39(5):805–17. 10.1007/s00296-019-04270-3. Epub 2019 Mar 12. PMID: 30864109. 30864109 10.1007/s00296-019-04270-3

[58] Albarracı D et al. (2005) ‘A test of Major assumptions about Behavior Change: a Comprehensive look at the effects of Passive and active HIV-Prevention interventions since the beginning of the epidemic’, 131(6), pp. 856–97. 10.1037/0033-2909.131.6.856 10.1037/0033-2909.131.6.856 PMC2713787 16351327

[59] Craig P, et al. Developing and evaluating complex interventions: the new Medical Research Council guidance’, *International journal of nursing studies*. Elsevier Ltd. 2008;50(5):587–92. 10.1016/j.ijnurstu.2012.09.010. 10.1016/j.ijnurstu.2012.09.010 23159157

[60] Michie S, van Stralen MM, West R. The behaviour change wheel: a new method for characterising and designing behaviour change interventions. Implement Sci. 2011;6:42. 10.1186/1748-5908-6-42. 21513547 10.1186/1748-5908-6-42 PMC3096582

[61] Michie S, Richardson M, Johnston M, et al. The behavior change technique taxonomy (v1) of 93 hierarchically clustered techniques: building an International Consensus for the reporting of Behavior Change interventions. Ann Behav med. 2013;46:81–95. 10.1007/s12160-013-948. 23512568 10.1007/s12160-013-9486-6

