# Supplementary material for: Effectiveness of behaviour change techniques in lifestyle interventions for non-communicable diseases: an umbrella review
Source: BMC Public Health. 2024 Nov 7;24:3082. doi: 10.1186/s12889-024-20612-8 (PMC11545567; doi:10.1186/s12889-024-20612-8)
Supplement: Supplementary file 1 — Supplementary Material 1 [file 12889_2024_20612_MOESM1_ESM.docx]

**Supplementary Table 1: Search Strategy**

| .no. | Search terms |
| --- | --- |
|  | ((behavio?r change technique* or behavio?r change therap* or behavio?r change model* or psychological technique* or psychological therap* or psychological model*) **AND** non-communicable disease*) or ncd or noncommunicable disease* or Cardiovascular Disease* or Heart Disease* or Myocarditis or Cardiomyopath* or Arrhythmias, Cardiac or Angina Pectoris or Myocardial Infarction or Coronary Disease or Hypertension or Ischemia or Cerebrovascular Disorders or Stroke or Hypercholesterolemia or Blood Pressure or Cholesterol or Blood Glucose or diabetes mellitus or diabetes or blood serum glucose or glucosaemia or glycaemia or Obesity or Neoplasm* or cancer or malignancy or tumor* or Asthma or Pulmonary Disease, Chronic Obstructive or obstructive pulmonary disease or obstructive pulmonary disorder or obstructive respiratory disease or Bronchitis or Lung Disease* or pulmonary disease or pulmonary disorder or chronic kidney disease* or chronic renal disease* or chronic nephropathy or kidney chronic failure or Liver Disease* or hepatic disease or hepatic disorder or Liver Cirrhosis or Osteoporosis or age related bone loss or Fibromyalgia or Musculoskeletal Disease* or orthopaedic disorder or Arthritis or Cystic Fibrosis or Endocrine System Disease* or endocrine disease or hormonal disorder or hormone dysfunction or hormone imbalance or Thyroid Disease* or Nervous System Disease*)) . |
|  | limit 1 to (full text and yr="1946 -Current") |
|  | limit 2 to "systematic review" |
