# Supplementary material for: Effectiveness of behaviour change techniques in lifestyle interventions for non-communicable diseases: an umbrella review
Source: BMC Public Health. 2024 Nov 7;24:3082. doi: 10.1186/s12889-024-20612-8 (PMC11545567; doi:10.1186/s12889-024-20612-8)
Supplement: Supplementary file 2 — Supplementary Material 2 [file 12889_2024_20612_MOESM2_ESM.docx]

**Supplumentary Table 2: Main characteristics and findings in the included systematic reviews**

| **No** | **Systematic reviews (***Author, article title, year, Country)* | **No. of primary studies included**  **AND**  **Countries** | **Design of primary studies** | **Condition** | **Quality Assessment (QA) scores** | **No. of pts** | **Intervention type** | **BCT labels and frequency of BCTs** | **Delivery mode/technique** | **Intervention duration and/or Follow up period** | **Main Findings – clinical and behavioural change outcome** |
| --- | --- | --- | --- | --- | --- | --- | --- | --- | --- | --- | --- |
| 1. | Heron et al. **Behaviour change techniques in home-based cardiac rehabilitation: a systematic review** 2016  UK | 11 studies  UK (4), Denmark (2), Australia (1), Canada (1), China (1), Norway (1), Poland (1) | RCTs | CVD | 10 (High) | 1,925 | Physical activity intervention  Any home-based cardiac rehab (CR) programme | **Total 20-**  Social support (unspecified) (11), goal setting (behaviour) (10), Reduce negative emotions (7), Instruction on how to perform the behaviour (7), Monitoring of behaviour by others without feedback (6), Self-monitoring of behaviour (6), Self-monitoring of outcome(s) of behaviour (6), Credible source  (6), Pharmacological support (6), Information about health consequences (5), Monitoring of outcome(s) of behaviour without feedback (3), Social support (practical) (3), Adding objects to the environment (3), Problem solving (2), Feedback on behaviour  (2), Biofeedback (2),  Information about emotional consequences (2), Demonstration of the behaviour (2), Behavioural practice/rehearsal (2), Feedback on outcome(s) of behaviour (1) | **Main intervention:**  Home-based cardiac rehab programme with use of manuals and  Internet  **Control group**  Hospital/l- or centre-based CR (7), received ‘usual care’ (2), received no active treatment1(2)  **Follow-up:** Telephonic counselling and/or monitoring | **Intervention duration**:  2 weeks - 12 months  **Follow-up period**:  2 -12 months | **Overall effect: (95%CI): NS**  All but one study reported the positive effect of home-based CR on modifiable CVD risk factors.  *Resting Systolic BP*: 1.02 mmHg**,** 1.74 to 3.78, *P* = 0.3  *Resting diastolic BP*: −0.89 mmHg, 4.35 to 2.58, *P* = 0.62  *Peak VO2max:* 1.19 ml/kg/min*,* 0.78 to 3.16, *P* = 0.24  *The distance covered in the 6-minute walk test*: 8.47 m,  10.98 to 27.92, *P* = 0.39  **Overall treatment effect (95% CI): NS**  *Total cholesterol*: 0.07 mmol/l, −0.16 to 0.29, P = 0.56  *HDL-cholesterol* : 0.01 mmol/l, 0.06 to 0.07, P = 0.79  *LDL-cholesterol*: 0.02 mmol/l,  0.25 to 0.29, P = 0.88  **Most BCT Used:**   - Social support - Goal setting (behaviour) - Feedback and monitoring.   ***Comment***: The BCT profile related to monitoring, instruction on how to perform the behaviour, and credible source were generally included in effective programmes |
| 2. | Duff et al.  **Behaviour change techniques in physical activity eHealth interventions for people with cardiovascular disease: systematic review**  2017  Ireland | 23 studies  USA (5), Australia (4), New Zealand (3) Canada (2), Norway (2), UK (2), Belgium (1), Italy (1), Korea (1), Poland (1), Taiwan (1) | RCTs | CVD | 7 (Medium) | 3,633 | Physical activity intervention | **Total – 30;** Information about health consequences (18), Goal setting (behaviour) (17), Self-monitoring of behaviour (11), Social support (practical) (11), Instruction on how to perform the behaviour (10), Feedback on behaviour (10), Biofeedback (9), Social support (unspecified) (9), Credible source (8), Self-monitoring of outcomes of behaviour (7), Action planning (7), Graded tasks (6), Feedback on outcome(s) of behaviour (5), Monitoring of outcomes of behaviour without feedback (5), Problem solving (4), Reduce negative emotions(4), Prompts/cues (3), Review behaviour goals (3), Goal setting (outcome) (3), Monitoring of behaviour by others without feedback (2), Social support (emotional) (2), Non-specific reward (1), Restructuring the physical environment (1), Adding objects to the environment (1), Pharmacological support (1), Demonstration of the behaviour (1), Social comparison (1), Review outcome goals (1), Social reward (1), Behavioural contract (1) | **Main intervention:** delivered via a computer, mobile phone, tablet, or phone (eg, mobile phone app, emails, text messages, and phone calls)  **Follow-up:** Community follow-up, drop-in sessions, phone support, visits to the patient's general practitioner | **Follow-up**:  3 weeks to 16 months | **Overall effect:**  Statistically significant improvements in PA between the experimental and control groups in eight of the 15 interventions.  **Most BCT Used:**   - Goal setting (behaviour) - Information about health consequences   ***Comment:***  Interventions not demonstrating a significant increase in PA (n=5) at par with the level achieved in standard cardiac rehab, with no significant differences between the control and experimental groups  Average number of BCTs used across significant interventions did not differ. The studies that increased PA versus those that did not increase PA both employed an average of seven BCTs. |
| 3. | van Vugt M et al**.**  **Use of behavioral change techniques in web-based self-management programs for type 2 diabetes patients: systematic review**  2013  Netherlands | 13 studies  USA (10), Korea (1), Netherlands (1), Norway (1) | RCTs (13) | T2DM | 7 (Medium) | 3,813 | Adherence to treatment intervention | **Total – 33,** Provide feedback on performance (8), Provide information on consequences of behaviour in general (7), Barrier identification/problem solving (7), Provide information on consequences of behaviour to the individual (6), Prompt self-monitoring of behaviour (6), Prompt self-monitoring of behavioural outcome (6), Provide instruction on how to perform the behaviour (5), Facilitate social comparison (5), Plan social support/social change (5), Goal setting (behaviour) (4), Action planning (4), Prompt review of behavioural goals (4), Stress management/emotional control training (4), Provide normative information about others’ behaviour (3), Model/Demonstrate the behaviour (3), Prompt practice (3), Use of follow-up prompts (3), Goal setting (outcome) (2), Provide rewards contingent on successful behaviour (2), Relapse prevention/coping planning (2), Provide information about others’ approval (1), Set graded tasks (1), Prompt review of outcome goals (1), Prompt rewards contingent on effort or progress toward behaviour (1), Prompting generalization of a target behaviour (1), Provide information on where and when to perform the behaviour (1), Teach to use prompts/cues (1), Environmental restructuring (1), Prompt identification as role model/position advocate (1), Prompt self-talk (1), Prompt use of imagery (1), General communication skills training (1), Stimulate anticipation of future rewards (1)  **The theories and models used:** self-efficacy theory (3), social cognitive theory (3), social-ecological (3), social support theory (1), transtheoretical model (TTM) (1), and cognitive behavioural therapy (1). | Online self-management diabetes programme | **Intervention duration**  1-18 months | **Overall effect:**  *Improvements in health behaviours outcomes*: Seven of 13 RCTs reported statistically significant (diet, physical activity/exercise, medication use, smoking)  *Clinical outcomes measures (glycated hemoglobin (HbA1c), fasting blood glucose, cholesterol, and triglycerides):* Nine studies reported statistically significant improvements.  *Psychological outcomes (depression, diabetes distress, psychosocial well-being, self-efficacy, stress, and communication):* Nine studies reported statistically significant improvements.  **Most BCT Used:**   - Feedback on performance, - Providing information on consequences of behaviour, - Barrier identification/problem solving, - Self-monitoring of behaviour.   ***Comment:***  These BCTs were also linked to positive outcomes for health behaviour change, psychological well-being, or clinical parameters. |
| 4 | McCullough et al**. Behaviour change theory, content and delivery of interventions to enhance adherence in chronic respiratory disease: a systematic review**  2016  UK | 46 studies  (Countries were not mentioned in the systematic review) | RCTs | Sleep apnoea; asthma; COPD | 8 (Medium) | 12,415 | Adherence to treatment | **Total Behaviour change/psychological theories** **- 12** Social cognitive theory (5), “Theory-based” but specific theory not reported (2), Self-efficacy theory (2), Compliance therapy model (1), Decisional balance (1), Health Belief Model (1), Horne and Weinman’s Benefit-risk model (1), Patient navigator model (1), Prospect theory (1), Protection motivation theory (1),  Transtheoretical model (1), Triandis theory of behaviour (1), | Face to face, Telephone, Internet, Unknown  One to one,  Group; Unknown | **Intervention duration**  One session of 1 hour to 6 months  **Intervention**  **Follow up**  28 days to 12 months | ***Comment:***  Most (n = 39, 67%) interventions were not based on behaviour change theory.  Nineteen interventions (33%) (from 15 studies) were designed using 12 different behaviour change theories.  A higher proportion of effective interventions (n = 11, 41%) used behaviour change theory to design their intervention than ineffective interventions (n = 8, 26%) |
| 5 | Avery el al.  **Changing physical activity behaviour in type 2 diabetes: a systematic review and meta-analysis of behavioural interventions**  2012  UK | 17 studies  Belgium (3), Canada (3), Italy (3), Australia (2), Netherlands (2), UK (2), Denmark (1), Korea (1) | RCTs | Type 2 diabetes | 9 (high) | 1,975 | Physical activity intervention | **Total: Psychological theories -** 5 - Social Cognitive Theory (7), Transtheoretical Model (5), Cognitive Behavioural Therapy (3), Motivational Interviewing  (3), Precede/Proceed Model (1),  **BCTs – 25;** Goal setting (17), Use of follow-up prompts (16), Prompt self-monitoring of behaviour  (16), Barrier identification/problem solving (15), Provide instruction on how to perform the behaviour (15), Prompt review of behavioural goals (14), Plan social support/social change (13), Relapse prevention/coping planning (11), Provide information on consequences of behaviour in general  (10), Set graded tasks (10), Provide information on where and when to perform the behaviour (10), Time management  (8), Provide feedback on performance (7), Action planning (7), Provide information on consequences of behaviour to the individual (5), Prompting generalisation of a target behaviour (5), Prompting focus on past success (4), Teach to use prompts/cues (4), Goal setting (outcome) (3), Prompt rewards contingent on effort or progress towards behaviour  (3), Motivational interviewing (3), Prompt self-monitoring of behavioural outcome (2), Provide rewards contingent on successful behaviour (1), Model /demonstrate the behaviour (1), Prompt practice (1) | Individual face to face sessions (12), group sessions (6), telephone (1) | **Intervention duration** –  8 weeks to 2 years | **Overall effect**  ***PA and* exercise - SS**  *Standardized mean difference* [SMD] = 0.45,  95% CI = 0.21–0.68, I2 = 55%  ***HBA1c : SS***  Weighted mean difference [WMD= −0.32%,  95% CI = −0.44 to –0.21%,  I2 = 8%  ***Changes in BMI: SS***  WMD = −1.05 kg/m2,  95% CI = −1.31 to −0.80,  I2= 2%  **Most BCT Used:**   - Prompting generalization of a target behaviour; - use of follow-up prompts; - Prompt review of behavioural goals - Provide information on where and when to perform PA - Plan social support/social change; - goal setting (behaviour) - Time management; - Prompting focus on past success; - Barrier identification/problem-solving; - Providing information on the consequences specific to the individual.   ***Comment:***  Clinically significant improvements in HbA1c were also suggested for studies utilizing more BCT median ≥10), interventions underpinned by a theory or model of behaviour change, and durations of ≥6 months.  Analyses suggest that utilization of above 10 different BCTs within behavioural interventions may be associated with clinically significant improvements in HbA1c (≥0.3% HbA1c): |
| 6 | Stacey et al.  **A systematic review and meta-analysis of social cognitive theory-based physical activity and/or nutrition behaviour change interventions for cancer survivors**  2015  Australia | 18 studies  USA (12),  Australia (1), Taiwan (1), unclear (4) | RCTs | Cancer  (at any point from diagnosis) | 7 (medium) | 2,765 | Dietary and physical activity intervention | **Theories:**  Social cognitive theory (5);  the transtheoretical model; stages of change (4)  **Total 21** –  Prompt self-monitoring of behaviour 27  Prompt self-monitoring of behavioural outcome 21  Plan social support/social chan(20)ge 18  Model/demonstrate the behaviour 18  Provide instruction on how to perform the behaviour 17  Prompt practice 17  Use of follow-up prompts 17  Goal setting (behaviour) 13  Barrier identification/problem solving 11  Action planning 9  Goal setting (outcome) 8  Prompt review of behavioural goals 8  Prompt review of outcome goals 8  Teach to use prompts/cues 5  Environmental restructuring 5  Facilitate social comparison 5  Relapse prevention/coping planning 5  Prompt anticipated regret 4  Provide information on consequences of behaviour in general 4  Provide information on consequences of behaviour to the individual 4  Prompting focus on past success 1 | Telephone counselling, face to face sessions, newsletter, written materials, face to face, facebook | **Intervention duration**  One session -18 weeks  **Intervention Follow up**  24 hour to 2 year | **Overall effect**  Significant improvements in one or more aspects of diet quality, over the medium to long term (6 months to 2 years) (5).  Nonsignificant decreases in energy and fat intake (2)  At 12 months, four trials reported improvements in PA, ranging from 20 min per week (P = 0.02) (1) to 89 min per week [(1) to 117 min per week (1), and a difference of 17.8 (P = 0.002) on the Leisure Score Index (1)  In PA-only trials, improvements in self-efficacy were associated with increased PA (3)  **Most BCT Used:**   - Goal setting.   **Most common strategy for self-efficay:**   - provide a pedometer - and/or a log sheet for self-monitoring of PA behaviour.   **Most common outcome expectancy target:**   - Social support or social comparison   ***Comment:***  Supports the Efficacy of SCT-based interventions in changing PA and diet behaviour in cancer survivors. Small-to-medium effect size 0.33 for PA interventions. |
| 7 | Eisele et al.  **Behaviour change techniques applied in interventions to enhance physical activity adherence in patients with chronic musculoskeletal conditions: a systematic review and meta-analysis** 2019  Germany | 22 studies  (Countries were not mentioned in the systematic review) | RCTs  (cluster-RCTs or quasi-RCTs) | Chronic musculoskeletal (CMC) | 10 (High) | 3,715 | Physical activity intervention | **Total 42:**  Instruction on how to perform a behaviour (38),  Information about health consequences (36),  Behavioural practice/ rehearsal (30).  Social support (practical) (29),  Demonstration of the behaviour (26),  Credible source (19),  Graded tasks (18),  Feedback on behaviour (16),  Action planning (15),  problem solving (14),  Goal setting (behaviour) (13),  Review behaviour goal(s)(7),  Adding objects to the environment (4),  Goal setting (outcome) (3),  commitment (3),  Feedback on outcome(s) of behaviour (3),  Generalisation of a target behaviour (3),  Social incentive (3),  Review outcome goal(s) (2),  Information about antecedents (2),  Behavioural experiments (2),  Monitoring of emotional consequences (2),  Prompts/cues(2),  Pros and cons (2),  Material reward (behaviour) (2)  Self‐reward (2),  Future punishment (2),  Focus on past success (2)  Discrepancy between current behaviour and goal (1),  Monitoring of behaviour by others without feedback (1),  Self‐monitoring of outcome(s) of behaviour (1),  Monitoring outcome(s) of behaviour by others without feedback (1),  Social support (emotional)(1),  Salience of consequences (1),  Information about social and environmental consequences (1),  Social comparison (1),  Exposure (1), ,  Habit reversal (1),  Non‐specific reward (1),  Pharmacological support (1),  Reduce negative emotions (1),  Identity associated with changed behaviour (1), | Face to face behavioural counselling, Education, exercise sessions, telephone, brochure/educational materials | **Intervention duration**  Single date to 12 months  **Intervention follow-up**  3 - 34 months | **Overall effect**  Nine interventions in nine studies were considered effective.  Significant between-group differences in the PA outcome (8),- sin favour of the intervention -7, in favour of the control group - 1.  d ≥0.5 or OR ≥ 2 in favour of the intervention group (2)  8 to 18 BCTs in intervention groups and 1 to 12 BCTs in control groups.  Small to medium-term effect of behaviour change interventions on PA adherence, but no long-term effect.  Interventions using a greater number of BCTs (between-group difference ≥8 BCTs) attained a higher effect than interventions applying a lower number of BCTs (between-group difference <8 BCTs).  **Most BCT Used:**   - Graded tasks - Goal setting - Self-monitoring - Problem solving - Feedback.   ***Comment:***  Superiority of single BCTs could not be shown.  The narrative analysis did not show superiority of any BCT, as all BCTs were used in both effective and non-effective interventions. |
| 8 | Yvonne et al., Effective behaviour change techniques in smoking  cessation interventions for people with chronic  obstructive pulmonary disease: A meta-analysis  2017. Effective behaviour change techniques in smoking  cessation interventions for people with chronic  obstructive pulmonary disease: A meta-analysis  Effective behaviour change techniques in smoking  cessation interventions for people with chronic  obstructive pulmonary disease: A meta-analysis  Effective behaviour change techniques in smoking  cessation interventions for people with chronic  obstructive pulmonary disease: A meta-analysis  Effective behaviour change techniques in smoking  cessation interventions for people with chronic  obstructive pulmonary disease: A meta-analysis  Barlett et al.  **Effective behaviour change techniques in smoking cessation interventions for people with chronic obstructive pulmonary disease: a meta-analysis**  2017  UK | 17 studies  Netherlands(4), USA (4), Denmark (3), Sweden (2), UK (2), Australia (1), multiple countries (USA, Spain, France, Italy) (1) | RCTs | COPD | 7 (Medium) | 7446 | Smoking cessation intervention  Linking COPD and smoking consultations, | **Total 47**  Boost motivation and self-efficacy (12)  Assess current and past smoking behaviour; (11)  Facilitate relapse prevention and coping (10)  Facilitate goal setting (10)  Advise on stop smoking medication (10)  Tailor interactions appropriately(10)  Provide information on the health consequences of smoking and smoking cessation 8  Facilitate action planning/ develop a treatment plan (8)  Give options for additional and later support (8)  Offer/direct towards appropriate written materials; (8)  Assess current readiness and ability to quit ; (8)  Facilitate barrier identification and problem solving (7)  Advise on/facilitate use of social support (7)  Adopt appropriate local procedures to enable clients to obtain free medication (7)  Emphasise choice (6)  Provide information on withdrawal symptoms; (6)  Provide feedback on current behaviour and progress (5)  Elicit client views (5)  Conduct motivational interviewing (4)  Identify reasons for wanting and not wanting to stop smoking (4)  Prompt review of set goals (4)  Build general rapport (4)  Explain expectations regarding treatment programme (4)  Assess nicotine dependence; (4)  Assess physiological and mental functioning (4)  Provide rewards contingent on not smoking (3)  Prompt commitment from the client there and then (3)  Provide rewards contingent on effort or progress (3)  Advise on environmental restructuring (3)  Advise on methods of weight control (3)  Ask about experiences of stop smoking medication that the smoker is using (3)  Assess past history of quit attempts (3)  Assess attitudes to smoking (3)  Explain the importance of abrupt cessation (2)  Measure carbon monoxide (CO) (2)  Advise on avoidance of social cues for smoking (2)  Elicit and answer questions (2)  Provide reassurance;.(2)  Explain how tobacco dependence develops (2)  Strengthen ex-smoker identity (1)  Advise on changing routine (1)  Advise on conserving mental resources (1)  Teach relaxation techniques (1)  Explain the purpose of carbon monoxide monitoring (1)  Use reflective listening (1)  Summarise information / confirm client decisions (1)  Assess number of contacts who smoke (1) | Smoking cessation using both advice and medication for people with COPD. 70.59% delivered in the home and 35.29% delivered exclusively in a clinical setting.  Interventions were one-to-one (70.59%) or a mixture of both one-to-one and group delivery (29.41%). | **Intervention duration**  22 days to 5 years  **Intervention**  **Follow-up**  Imminent to 2 years  (after all active components of the intervention had stopped) | **Overall effect**  Sample-weighted average quit rate was 13.19%.  Interventions that provided smoking cessation medication as a mandatory part of their protocol (k = 7, d+ = 0.42) were more effective than interventions that did not.  Interventions delivered exclusively in a clinical setting (k = 6, d+ = 0.37) had a significantly higher d+ than those that contained either home components, or were delivered exclusively at home.  Interventions that deployed *Facilitate action planning/develop treatment plan, Prompt self-recording, Advise on methods of weight control, and Advise on/facilitate use of social support* each engendered significantly larger effect sizes compared with studies that did not use these techniques.  The provision of COPD-specific information or COPD medication  advice was not associated with effect sizes respectively. However, interventions that involved linking COPD and smoking  generated larger effect sizes (Qb = 8.42, p < .01). |
| 9 | Cradock et al.  **Behaviour change techniques targeting both diet and physical activity in type 2 diabetes: A systematic review and meta-analysis**  2017  Ireland | 13 studies  USA (5), Korea (3), Australia (2), Finland (1), Germany (1), UK (1) | RCTs | Type 2 diabetes | 8 (medium) | 6,639 | Dietary and physical activity intervention | **Total - 46**  Instruction on how to perform a behaviour (13),  Action planning (12),  Credible source(12),  Goal setting (behaviour) (11), Goal setting (outcome) (10), Social support (unspecified) (10),  Self-monitoring of behaviour (9), Feedback on behaviour (7),  Demonstration of the behaviour (7), Graded tasks (7), Adding objects to the environment (7), Problem solving (5),  Monitoring outcome(s) of behaviour by others without feedback (5),  Behavioural practice/rehearsal (5),  Avoidance/reducing exposure to cues for the behaviour (4), Review behaviour goal(s) (3),  Review outcome goal(s) (3), Self-monitoring of outcome(s) of behaviour (3), Feedback on outcome(s) of behaviour (3), Restructuring the physical environment (3),  Monitoring of behaviour by others without feedback (2), Social support (emotional) (2),  Information about health consequences (2),  Social comparison (2),  Prompts/cues (2),  Behaviour substitution (2),  Generalization of a target behaviour (2),  Non-specific reward (2),  Self-reward (2),  Self-talk (2),  Discrepancy between current behaviour and goal (1), Biofeedback (1),  Social support (practical) (1), Remove aversive stimulus (1),  Habit formation (1),  Pros and cons (1), Material reward (behaviour) (1),  Social reward (1),  Non-specific incentive (1),  Self-incentive (1),  Reduce negative emotions (1), Restructuring the social environment (1),  Identification of self as role model (1),  Framing/reframing (1),  Verbal persuasion about capability (1),  Focus on past success (1) | Individual sessions  & group sessions where participants meet face to face with interventionist/dietician, trainers  Workbook (1), telephone calls (4) | **Intervention duration**  12 weeks to 10 years | **Overall effect**  *Reductions in HbA1c at:*  *3 months:* -1.11 % (12 mmol/mol)  *6 months:* -0.67 % (7 mmol/mol)  *12 months:* -0.28 % (3 mmol/mol)  *24 months*: -0.26 % (2 mmol/mol) *Overall reduction* : -0.53 %  6 mmol/mol (95 % CI -0.74 to -0.32, P < 0.00001).  *Reduction in body weight* :  *3 months*:-2.7 kg  *6 months*: -3.64 kg  *12 months*: -3.77 kg  *24 months*: -3.18 kg  *Overall reduction*: -3.73 kg  (95 % CI -6.09 to -1.37 kg, P = 0.002).  **Most BCT associated with Improvements of outcomes (4/56):**   - instruction on how to perform a behaviour - behavioural practice/rehearsal - demonstration of the behaviour - action planningand   **Most Common intervention features:**   - Supervised physical activity - group sessions - contact with an exercise physiologist - contact with an exercise physiologist and a dietitian - baseline HbA1c >8 %’   ***Comment:***  Interventions of greater frequency and intensity were associated with clinically significant reductions in HbA1c (> 0.3 %). |
| 10 | Fredrix et al. **Goal setting in diabetes self-management: A systematic review and meta-analysis examining content and effectiveness of goal-setting interventions**  2018  Ireland | 14 studies  USA (11), Netherlands (2), UK (1) | RCTs; pre and post design. | Diabetes Mellitus  T1DM or T2DM | 10 (High) | 3,015 | Dietary and physical activity intervention  Goal setting and action planning) education, counselling sessions, within diabetes self-management programmes/ targeting diabetic control. | **Total 10**  Goal-setting (behaviour) (10) Problem solving (8)  Action planning (7)  Review outcome goal(s) (5)  Review behaviour goal(s) (4) Biofeedback (4)  Goal-setting (outcome) (3)  Self-monitoring of behaviour (2)  Assessing knowledge attitude and ambivalence (1) Counselling to provide goal feedback (2) | **Main intervention:**  Individual and group face to face sessions only; telephone only; Mixture of face to face and online; Computer guided by telephone.  Within the home, medical setting and community centre.  **Follow up**  Mainly phone call only | **Intervention duration**  1 week to 24 weeks.  **Follow up** between 3 to 24 weeks. | **Overall effect**  ***blood glucose levels (HbA1c):SS***  A meta-analysis demonstrated a significant decrease (p = .02) in average. Funnel plot for (a) Blood glucose levels and (b) Body weight. Psychology & Health 967 of −.22 (95% CI, −.40, −.04). An I 2 of 22% was calculated, indicating a low percentage of variation across study results due to heterogeneity rather than chance.  ***Body weight****:* ***NS***  Mean Difference: .06, 95% CI −1.17 to 1.28, p = .96)  I2: 0%  ***Self-efficacy***  93% of patients achieved at least one of their goals during the intervention.  **Most BCTs used (10/12 or 83.3%):**   - Goal-setting (behaviour)   ***Comment:***  Review outcome goals’ was used most frequently per intervention, and was coded an average of 2.6 times within each intervention. |
| 11 | Hall  Hallward et al. **Behaviour change techniques in physical activity interventions for men with prostate cancer: a systematic review**  2020  Canada | 15 studies  Canada (7), Australia (3), USA (3), Spain (1), Sweden (1) | RCTs and Quasi experiments; pre experimental designs | Cancer  various stages of prostate cancer, | 6 (Medium) | 2,274 | Physical activity.  Ranging from aerobic training, resistance training and yoga | **Total 21**  Goal setting (behaviour) (2)  Problem solving (2)  Action planning (1)  Review behaviour goals (1)  Feedback on behaviour (1)  Self-monitoring of behaviour (9)  Biofeedback (4)  Social support (unspecified) (8)  Social support (practical) (3)  Instruction on how to perform the behaviour (11)  Information about antecedents (3)  Information about social and environmental consequences (5)  Demonstration of the behaviour (6)  Prompts and cues (3)  Behavioural practice/rehearsal (15)  Generalization of target behaviour (8)  Graded tasks (7)  Credible source (3)  Material reward (behaviour) (1)  Non-specific reward (1)  Adding objects to the environment (9) | Face to face and telephone calls encouraging weekly physical activity with booster sessions to increase adherence to the programme.  Nine studies had control groups that were subjected to usual care, received information regarding physical activity and health or practised light stretching.  Interventions were conducted in a gym or exercise clinic (n=1), were strictly home-based (n=3) or were both clinic and home-based (n=11). All of the interventions implemented in clinics were supervised by an accredited exercise physiologist or kinesiologist | **Follow up**  1 to 12 months | **Overall effect**  Studies classified into four categories based on the promise of the intervention to change physical activity behaviour.  Five interventions (33.33%) were classified as very promising, one intervention (6.67%) as quite promising and the other interventions, non-promising or unclear.  The six promising studies showed significant increases in physical activity levels postintervention.  **Most promising BCTs:**   - social support (practical; ratio=2), - information about antecedents (ratio=3), - information about social and environmental consequences (ratio=2), - prompts and cues (ratio=2), - credible source (ratio=2) - adding objects to the environment (ratio=2.5).   ***Comment:***  Social support (practical), information about antecedents and prompts and cues appeared in promising interventions and not in non-promising interventions.  Information about antecedents is the one BCT that appeared only in promising interventions and not in interventions classified as non-promising or unclear promise. |
| 12 | E  Evangelidis et al. **Lifestyle behaviour change**  **for preventing the progression of chronic kidney disease: a**  **systematic review** 2019  Australia | 26 studies  US (5), Brazil (3), Australia (2), Canada (2), Netherlands (2), Taiwan (2), UK (2), Algeria (1), Belgium (1), China (1), France (1), India (1), Italy (1), Japan (1), Thailand (1) | RCTs | Chronic Kidney Disease  stages 1 to 5 | 8 (Medium) | 4,263 | Mix of interventions.  A mixture of counselling, advise, Dietary only; physical activity only; weight reduction and/or smoking cessation. | **Total 39**  Instruction on behaviour 23  Social support (unspecified) 16  Demonstration of the behaviour 13  Feedback on behaviour 12  Behavioural practice/rehearsal 12  Goal setting (behaviour) 8  Review behaviour goal(s) 8  Review outcome goal(s) 8  Self-monitoring of behaviour 8  Graded tasks 8  Problem solving 7  Behaviour substitution 6  Goal setting (outcome) 5  Monitoring of behaviour by others without feedback 5  Information about health consequences 5  Habit reversal 5  Adding objects to the environment 5  Action planning 4  Behavioural contract 4  Social support (emotional) 4  Self-monitoring of outcome(s) of behaviour 3  Generalisation of target behaviour 3  Pros and cons 3  Biofeedback 2  Social support (practical) 2  Commitment 1  Feedback on outcome(s) of behaviour 1  Behavioural experiments 1  Salience of consequences 1  Monitoring of emotional consequences 1  Social comparison 1  Prompts/cues 1  Non-specific reward 1  Social reward 1  Reward (outcome) 1  Reduce negative emotions 1  Conserving mental resource 1  Verbal persuasion capability 1  Focus on past success 1 | Group as well as individual educational and counselling, and support programmes within range of settings from home, gym, hospital, rehab facility | **Intervention duration**  1 to 45 months | **Overall effect**  *Significant Improvement in primary outcome (69% or 18 studies):*   - *eGFR* - *blood pressure,* - *peak VO2* - *sodium or albumin excretion*   *No meta-analysis of the data possible due to heterogeneity of outcome measures*.  **Most common strategies:**   - *Education , 21 (81%) interventions.* - *Enablement/empowerment to improve self-management of diet, lifestyle and physical activity, 18 (69%) of interventions.* - *Training, 12 (46%) interventions*   **Least common strategies**   - Family support   ***Comment:***  Optimising the social environment and arranging support from friends, family and the community may improve lifestyle behaviour change interventions for patients with CKD |
| 13 | Lee et al.  **A systematic review of the effectiveness of problem-solving approaches towards symptom management in cancer care**  2010  Taiwan | 7 studies  USA (7) | RCTs; controlled clinical trials and quasi-experimental studies design | Cancer  (different types of cancer e.g. breast, prostate, and lung) | 6 (medium) | 1,608 | Adhering to treatment | **Total 4**  Problem solving (7)  Social support (emotional) (1)  Social support (practical) (1),  Information about health consequences (1) | Face-to-face or telephone counselling and education.  Materials such as booklets and videotapes were also provided. | **Intervention duration**  30 minutes to 20 weeks  **Follow up**  16 days to 32 weeks | **Overall effect**  **Improvement of symptoms of**   - pain, fatigue distress - decrease in symptom intensity at 10- and 20- week follow-up for younger patients (5 contacts out 8 weeks). - Symptom distress at a 30- day follow-up (3 out of 9 contacts). - decreased number of symptoms post intervention (2 studies)   **Most effective approach to reduce impact of symptoms and develop coping tsrategies**   - problem-solving approach (5 out 7 studies). |
| 14 | sssssfgffffadnhhg  Igwesi-Chidobe et al. **Physical activity containing behavioural interventions for adults living with modifiable chronic non-communicable diseases in Africa:**  **a systematic mixed-studies review**  2018  South Africa | 6 studies  South Africa (4),  Reunion Island (2) | RCT, mixed method; non control; qualitative studies. | Type 2 Diabetes mellitus | 8 (High) | 517 | Physical activity  intervention  (combined psychoeducational sessions, with mobile phone-based peer support) | **Total 19**  Shaping knowledge (information provision) (3),  restructuring the physical and social environment (1),  behavioural rehearsal/practice (1),  habit formation(1),  self-monitoring of behaviour (2),  goal setting (behaviour) (3),  feedback on behaviour(2),  problem-solving (3),  action planning (3),  health consequences (3),  regulate negative emotions (2),  prompts/cues (2),  action planning (including implementation intentions) (2)  identification of self as role model (1),  pharmacological support (1),  social support (practical) (1),  social support (general) (1),  social support (emotional) (1),  instruction on how to perform a behaviour (1) | Face-to-face, group-based; telephone based educational programmes within primary and tertiary settings | **Intervention duration**  4 weeks to 9 months  **Follow up**  6 to 12 months | **Overall effect**  Overall improvement   - Exercises and sports activities increased in the short term - Conflicting evidence on the effects of interventions on home and occupational physical activities.   ***Comment:***  This review could not confirm the BCTs responsible for improvements in physical activity because primary clinical studies did not investigate this. |
| 15 | Ismail et al. **Systematic review and meta-analysis of randomised controlled trials of psychological interventions to improve glycaemic control in patients with type 2 diabetes** 2004  UK | 25 studies  USA (15), Australia (4), China (2), Canada (1), Japan (1), Spain (1), UK (1) | RCTs | Type 2 Diabetes mellitus/ | 10 (high) | 1,527 | Adhering to treatment  Psychological interventions for improving control of diabetes with a control group of usual care, education, waiting list, or attention control | **Total = 10**  (converted Michie’s taxonomy)  Behavioural experiments (24) Instruction on how to perform the behaviour (15)  Behaviour substitution (6)  Behavioural practice/rehearsal (6)  Motivational interviewing (3) Pharmacological support (3) Self-monitoring of behaviour (2) Social support (practical) (1)  Mental rehearsal of successful performance (1)  Goal setting (behaviour) (1) | Face to face and group Cognitive behaviour therapy, education and counselling sessions | **Intervention duration**  1 week to 6 months  **Follow up.**  1 to 12 months | **Overall effect**  Significant improvement   - glycaemic control (absolute difference of 0·76% in glycated haemoglobin) with psychological therapies. - pooled effect size was larger, representing a difference of 1·00% in glycated haemoglobin with less intensive psychological theray excluded.   **Most effective therapy:**   - Psychological therapies   **Most effective strategies using cognitive behaviour model:**   - relaxation techniques - problem solving, - contract setting, - goal setting, - self-monitoring of behaviours, and - enlisting social support. - counselling techniques   ***Comment:***  Psychological therapy was associated with a reduction in psychological distress but did not appear to affect weight control or blood glucose concentration. |
| 16 | Larkin et al.  **Behaviour change interventions to promote physical activity in rheumatoid arthritis: a systematic review**  2015  Ireland | 5 studies  (Countries were not mentioned in the systematic review) | RCTs | Rheumatoid arthritis | 6 (Medium) | 784 | Physical activity  intervention  consisted of aerobic, strengthening and range of motion exercises and were both land and water based; | **Total 21**  Persuasive argument (6)  goal setting (4)  Self-monitoring (4),  review of behaviour goals (4)  Pros and cons identification (4)  problem solving (3)  instruction on how to perform a behaviour (3)  coping planning (2)  self-monitoring of behaviour (2)  social support (2)  social support (practical)(2)  social support (general)(1)  behavioural rehearsal/practice (1)  others monitoring with awareness (1)  social comparison (1)  Graded tasks (1)  self-reward (1)  focus on past success (1),  action planning (1),  anticipation of future rewards (1)  prompts/cues (1) | Classed based one to one/group/telephone/internet-based intervention. | **Intervention duration**  8 week to 1 year  **Follow up**  12 month to 2 years | **Overall effect**  Not Significant   - Only short-term increases in physical activity behaviour (3 studies) - Maintanence in long term term ( 1 study) - no increase in physical activity behaviour post-intervention or at either shorter long-term follow-up (2 studies) |
| 17 | O’Dwyer et al. **Behaviour change interventions targeting physical activity in adults with fibromyalgia: a systematic review** 2019  Ireland | 8 studies  USA (4), Canada (1), Sweden (1), Switzerland (1), UK (1) | RCT and 1 quasi-RCT | Fibromyalgia | 7 (Medium) | 924 | Physical activity  intervention  aerobic, relaxation and home exercise programmes | In total, 32 different BCTs were identified within the eight studies. All studies ( n =8) incorporated ‘instruc - tion on how to perform the behaviour’, ‘demonstration of the behaviour’, ‘behavioural practice/rehearsal’, and ‘credible source’  (Did not indicated frequency of the 32 identified BCTs)  eHealth belief model, Pender’s model of health promotion and self-efficacy theory. Moti - vational Interviewing, social cognitive theory and the self-management cognitive-behavioural therapy approach | Interventions were primarily delivered in group settings (n=6), online, or by phone | **Intervention durations** 6 weeks to 16 weeks  **Follow-up** 3 months to 12 months | **Overall effect**   - short-term improvements in PA but no improvements being maintained at follow-up. - increases in PA behaviour at follow-up but not at the end of the intervention phase - no significant effects on PA behaviour post-intervention or at follow-up.   ***Comment:***  Differing behaviour change theories and techniques were implemented across interventions, the mode of implementing the intervention, the variety of PA outcome measures used, and the varying risk of bias across the studies may have contributed to inconsistent findings. |
| 18 | D'Egidio et al.,  **Counseling interventions delivered in women with breast cancer to improve health-related quality of life: a systematic review** 2017  Italy | 35 studies  (Countries were not mentioned in the systematic review) | 24 RCTs, two protocols of RCT, three cross-sectional studies, two narrative reviews, two systematic reviews, and one overview. | Cancer  Breast cancer | 6 (Medium) | Not mentioned | Diet and physical activity intervention | **Total 5**  Converted to Michie’s taxonomy  Social support (unspecified) (3)  Information about emotional consequences (11)  Behavioural experiments (6)  Social Support (emotional) (1)  Reduce negative emotions (1) | Telephone and face to face group CBT, educational, counselling, and health app | **Intervention durations**  1 month to  12 months  **Follow-up**  18 months | **Overall effect**   - Wellness modification interventions (All studies) - Lifestyle interventions can be conducted as single or combined counselling and can support physical functioning of cancer patients. - The combination of exercise and psychological counselling program seemed to be more effective than single ones, and feasible and acceptable, and improved some aspects of QOL, such as depression, but these benefits may diminish over time. - Another approach to deliver nutrition and exercise information was through a mobile-based application, which gave good results with moderate quality of evidence. - The efficacy of lifestyle interventions were increased by additional tools as pedometer, self-monitoring calendars for physical activity, booklet, DVD, mail information about topics discussed in the counselling sessions. |
| 19 | Dombrowski et al. **Identifying active ingredients in complex**  **behavioural interventions for obese adults with**  **obesity-related co-morbidities or additional risk**  **factors for co-morbidities: a systematic review** 2012  UK | 44 studies  USA (27), Canada (5), UK (5), Australia (4), Finland (2), Netherlands (1) | RCTs | Participants with a mean/median BMI≥30 and at least one additional risk factor for morbidity or an already present co-morbidity | 9 (High) | 10,055 | Diet and physical activity intervention | **Total 24**  Prompt intention formation 54  Provide opportunities for social comparison 52  Provide instruction 50  Prompt self-monitoring of behaviour 50  Prompt barrier identification 47  Plan social support/social change 41  Provide feedback on performance 28  Prompt practice 27  Relapse prevention 27  Provide information on consequences 26  Provide general encouragement 21  Provide general information 19  Set graded tasks 19  Provide contingent rewards 18  Prompt review of behavioural goals 14  Prompt specific goal setting 12  Stress management (stress theories) 12  Teach to use prompts/cues 9  Model/demonstrate the behaviour 8  Use follow-up prompts 6  Prompt self-talk 5  Motivational interviewing 5  Time management 5  Agree behavioural contract 1  Abbreviations:  IMB, Information-Motivation-Behavioural Skills model; TRA, Theory of Reasoned Action; TPB, Theory of Planned Behaviour; SCogT,  Social Cognitive Theory; CT, Control Theory; OC, operant conditioning; SCompT, theories of social  comparison. | Group format and individual format | **Intervention duration**  1 to 14 months  **Follow up**  ≥12 weeks | **Overall effect**   - The mean difference of weight loss between intervention and control groups = -3.0 kg (95%CI -4.3 to -1.8) - The mean difference of calorie intake between intervention and control groups = -112 kcal (95%CI -217 to -7) - The standardised mean difference between intervention and control groups = 0.3 (95%CI 0.2 to 0.5)   **Intervention delivery**   - Delivery format and timing of intervention period were not related to weight loss. - Contact frequency was related to weight loss (p = 0.005). - Recruitment setting associated with weight loss –   - community (-4.7 kg),   - general practice (-1.2kg),   - clinical settings (-0.3 kg)   - community vs clinical settings, p = 0.023;   - community vs general practice, p = 0.063.   **Effects of BCTs**  *Weight loss using and non-using BCTs for diet*   - Provide instruction: -4.3 kg vs -1.5 kg, p = 0.023 - Self-monitoring: -4.2 kg vs -0.8 kg, p = 0.005 - Relapse prevention: -4.5 kg vs -1.7 kg, p = 0.028   *Weight loss using and non-using BCTs for PA*   - General information: -0.7 kg vs -3.7 kg, p = 0.031 - Information on consequences: -1.2 kg vs -3.9 kg, p = 0.04 - Prompt practice: -4.8 kg vs -1.2 kg, p = 0.001   *Calorie intake using and non-using BCTs*   - Provide instruction: -196 kcal vs 71, p = 0.02 |
| 20 | Sherifali et al. **Evaluating the effect of a diabetes health coach in individuals with type 2 diabetes**  2016  Canada | 8 studies  USA (4), Australia (1), Finland (1), Korea (1), Turkey (1) | RCTs | Type 2 diabetes | 8 (Medium) | 724 | Adherence to treatment  (Medication and/treatment through health coaching ) | **Total = 4**  *Converted to Michie’s taxonomy*  Self-monitoring of behaviour (11)  Information about health consequences (8)  Feedback on outcome(s) of behaviour (8)  Goal setting (outcome) (6) | Face-to-face (1), telephone (4), combination of face-to-face and telephone (2), combination of face-to-face and internet (1) | **Intervention duration**  12 to 64 weeks  **Follow up**  12 weeks to 16 months | **Overall effect**  Significant effect   - pooled decrease in HbA1C levels of −0.32% (95% CI, −0.50 to −0.15), - with the greatest effects seen in long term coaching (>6 months) for A1C levels (−0.57%; 95% CI, −0.76 to −0.38). - short-term intervention (≤6 months) vs control = -0.23% (95%CI -0.37 to -0.09)   ***Comment:***  Recognizing that health coaching for those with diabetes comprises a variety of complex components and strategies, this review found that all coaching interventions included elements of goal setting, acquisition of knowledge about diabetes, individualized care and frequent follow up. |
| 21 | Meade et al. **Behaviour change techniques associated with adherence to prescribed exercise in patients with persistent musculoskeletal pain: systematic review** 2019  UK | 8 studies  Sweden (2), USA (2), Austria (1), China (1), Finland (1), UK (1) | RCTs | Persistent musculoskeletal pain (PMSK)  (Fibromyalgia, low back pain) | 7 (Medium) | 1,018 | Physical activity intervention  Biofeedback/ relaxation, Motivation enhancement, Goal-setting therapy, Neck-specific exercise and behaviour enhancement  therapy, Motivation enhancement and exercise programme, Back school with relaxation and counselling, Personalized and monitored exercise programme, Exercise and intensive counselling | **Total BCTs in treatment group = 28**:  Instruction on how to perform a behaviour (6),  Demonstration of behaviour (5),  Behavioural practice/rehearsal (5),  Goal setting (behaviour) (3),  Action planning (3),  Graded tasks (3),  Body changes (3),  Problem solving (2),  Review behaviour goals (2),  Discrepancy between current behaviour and goal (2),  Feedback on behaviour (2),  Social support (unspecified) (2),  Prompts/cues (2),  Goal setting (outcome) (1),  Review outcome goal(s) (1),  Behavioural contract (1),  Commitment (1),  Self-monitoring of behaviour (1),  Biofeedback (1),  Information about health consequences (1),  Monitoring of emotional consequences (1),  Generalization of a target behaviour (1),  Credible source (1),  Comparative imagining of future outcomes (1),  Nonspecific rewards (1),  Social reward (1),  Future punishment (1),  Framing/reframing (1) | At Clinic (5)  Local health club (2)  Not report (1) | **Intervention duration**  1-15 sessions (1-4* per week)  **Follow up**  15 days to 5 years | **Overall effect**  Significant effect on exercise adherance   - Eight RCTs (five low, three high RoB) met inclusion criteria. Five trials reported between-group differences   **Most effective BCT Used**   - *social support,* - *goal setting,* - *instruction of behaviour,* - *demonstration of behaviour,* - *practice/rehearsal,* - *improved exercise adherence.*   ***Comment:***  Three trials reported theoretical underpinning. Interventions employing ≤seven BCTs, unique to those included in the control group, were most effective at enhancing exercise adherence. |
| 22. | Grimmett et al. **Systematic review and meta-analysis of maintenance of physical activity behaviour change in cancer survivors** 2019  UK | 27 studies  USA (11), Australia (5), Canada (4), Netherlands (2), Belgium (1), Germany (1), Hong Kong (1), Ireland (1), UK (1) | RCTs | Cancer: Breast, prostate, colon and mixed | 8 (Medium) | 5,792 | Physical activity intervention | **Total 31**  Goal setting (behaviour) (25),  Self-monitoring of behaviour (22),  Instruction on how to perform a behaviour (19),  Credible source (19),  Problem solving (18),  Social support (unspecified) (18),  Adding objects to the environment (17),  information about health consequences (16),  Action planning (15),  Graded tasks (14),  Feedback on behaviour (11),  Verbal persuasion about capabilities (10)  Review behaviour goal(s) (8),  Prompts/cues (8),  Demonstration of behaviour (7),  Social reward (6),  Reduce negative emotions (6),  Generalisation of a target behaviour (5),  Behavioural practice/ rehearsal (4),  Goal setting (outcome) (3),  Discrepancy between current behaviour and goal (3),  Salience of consequences (3),  Pros and cons (3),  Social support (practical) staff (2),  Information about social and environmental consequences (2),  Information about emotional consequences (2),  Framing/reframing (2),  Commitment (1),  Biofeedback (1),  Feedback on outcome(s) of behaviour (1),  Information about antecedents (1), | Group supervised exercise classes and home-based exercise with referral to GP exercise scheme, Telephone delivered home-based, Supervised exercise sessions, counselling and group discussions. Home-based exercise encouraged, Printed 11 chapter guidebook to promoted PA tailored to young adults, Peer-led telephone consultation and mailed feedback reports, Health care professional advice plus telephone counselling plus printed materials, Residential rehab program with telephone follow-up, Web-based lifestyle intervention | **Intervention duration**  A single contact to 10 months  **Intervention follow-up**  3 months to 5 years. | **Overall effect**   - SMD in moderate to vigorous physical activity (MVPA) between groups 0.25; 95% CI = 0.16–0.35. - Within-group pre-post intervention analysis yielded a mean increase of 27.48 (95% CI = 11.48-43.49) mins/wk. of MVPA in control groups and 65.30 (95% CI = 45.59–85.01) mins/wk. of MVPA in intervention   groups.  **Characteristics ineffective interventions**   - included older populations with existing physical limitations, - had fewer contacts with participants, - were less likely to include a supervised element - did not include the BCTs of ‘action planning’, ‘graded tasks’ and ‘social support (unspecified)’.   ***Comment:***  Included studies were biased towards inclusion of younger, female, well-educated and white populations who were already engaging in some physical activity. |
| 23 | D  Denford et al. **Effective behavior change techniques in asthma self-care interventions: systematic review and meta-regression**  2014  UK | 38 studies  (Countries were not mentioned in the systematic review) | RCTs | Asthma | 8 (Medium) | 7,883 | Adherence to treatment  Interventions targeting asthma self-care behaviour contained one or more behaviour change technique | **Total - 18**  Self-monitoring (35)  Instruction (30),  Goal setting (26)  Inhaler technique (24),  Providing feedback on inhaler technique (24),  Medication optimization (11),  Use of active learning techniques (11),  Active involvement of participants (10),  Individual tailoring of information or of intervention content (9),  Addressing medication concerns (7),  Prompt specific goal setting (5), `  Prompt review of behavioural goals’ (5),  Provide feedback on performance (5)  Developing illness models (4) (i.e. improving understanding of the illness’ identity, cause, controllability, consequences and timeline)  prompt intention formation’(5),  Training in social communication skills (3),  Cognitive behaviour therapy (3),  Efficacy building (1),  Five of 38 interventions included all of the behaviour change techniques associated with Control Theory. | Interventions were delivered individually (30), in groups (1), in a combination of group and individual sessions (5) or were self-administered (2). | **Follow up:**  2 to 18 months | **Overall effect**  *Significant effect (38 trials meta analysis) of asthma self-care*   - reduction in symptoms and unscheduled health care use - increased adherence to preventive medication   *Significant effect (meta regression) of active involvement of particpants*   - reduction in unscheduled health care use (OR=0.50 vs. 0.79).   *Significant effect of stress management*   - increase in asthma symptoms (SMD= 0.01vs. -0.44).   ***Comment:***  **BCT effect**  *Reduction in asthma symptoms:*   - *Providing feedback:* somewhat significant association between interventions and symptom reduction - *Social Cognitive Theory or Control Theory* : not significantly associated with symptoms of asthma. - *Other BCT’s*: No significant associations   *Unscheduled health care use:*   - *behavioural theory*: approaching significance (p=0.5). - *Active involvement of participants*: significantly associated - *Medication concerns*: approaching significance. - Control Theory: not significant   *Adherence to medication:*   - no statistically significant associations (p >0 .05) - *behaviour change techniques:* no significant association - *Control Theory or Social Cognitive Theory* : not significant |
| 24 | Etminani et al.  **How behaviour change strategies are used to design digital interventions to improve medication adherence and blood pressure among patients with hypertension: systematic review**  2020  Sweden | 54 studies  (Countries were not mentioned in the systematic review)  North America (24), Europe (14), Asia (8), Latin America (4), Africa (2), Australia (2) | 39 RCTs, 5 longitudinal studies, 5 pilot/feasibility studies, 3 quasi-experimental studies, 1 observational study and mixed-method study. | Hypertension | 3 (Low) | 19 to 4076 participants. | Adherence to treatment  Medication and blood pressure monitoring. | **Total BCTs – 1**  Techniques targeting patients’ psychological determinants - Michie’s taxonomy - Monitoring of emotional consequences (4)  **Total Models and principles - 6**  The Chronic Care Model (4) the Social Cognitive Theory (4), the Health Belief Model (1), The Self-Determination Theory (2) Patient empowerment as underpinning cognitive model (2), Common-Sense Model of Self-Regulation (2)    *Only 19/54 studies reporting use of BCTs or theories/models* | Phone (23 studies): phone calls, including manual (19 studies), Interactive Voice Response (3 studies), and videoconferencing (3 studies) • Web (26 studies): Web-based platforms • SMS (13 studies) • mHealth (16 studies): mHealth smartphone apps • Email (16 studies) • Electronic health records (EHR; 7 studies): EHR-based software • Video (3 studies): non-Web-based educational multimedia content • Com (2 studies): computer-based programs • TelDev (8 studies): telemetric devices, including automatic BP monitoring devices or automatic weighing scales • ePill (6 studies) • Digital medicine ((2 studies) | Not mentioned | **Overall effect**   - *Tailored interventions* *addressing each patient’s specific barriers* to *adherence*: successful - *Digital* *interventions*: not successful - *Use of theoretical model:* successful   ***Comment:***  65% of the reviewed studies did not mention the use of theory, models, or principles in their intervention planning. |
| 25 | Winkley et al.  **Psychological interventions to improve glycaemic control in adults with type 2 diabetes: a systematic review and meta-analysis**  2020  UK | 94 studies  USA (32), Netherlands (9), UK (7), Germany (6), Iran (6), Australia (4), China (4), Denmark (3), Taiwan (3), Belgium (2), Canada (2), Italy (2), Malaysia (2), New Zealand (2), Brazil (1), Chile (1), Croatia (1), Finland (1), Ireland (1), Norway (1), Portugal (1), Spain (1) Thailand (1), Turkey (1) | RCTs  (70 RCTs had HbA1c data which could be pooled.) | Type 2 Diabetes | 10 (High) | 14,796 | Adherence to treatment  (Managing glucose control through counselling such as CBT, relaxation therapy) | **Total – 18**  *Converted to Michie’s taxonomy -*  Motivational interviewing (24)  Behavioural experiments (10)  Self-monitoring of behaviour (8)  Behavioural practice/rehearsal (6)  Information about health consequences (6)  Social support (practical) (5)  Social support (unspecified) (5)  Feedback on behaviour(4)  Instruction on how to perform the behaviour (4)  Feedback on behaviour (4)  Social support (emotional) (3)  Problem solving (3 )  Commitment (3)  Mental rehearsal of successful performance (3)  Habit formation (1)  Information about Antecedents (1)  Information about emotional consequences (1)  Imaginary reward (1) | Most interventions were delivered face to face (n=75), and mostly to individuals (n=54) and groups (n=37). Twenty-seven studies referred to an intervention manual, of which 7 provided a link to the manual and 24 studies provided a link to the study protocol. | **Follow Up:**  12-month | **Overall effect**   - *psychology professionals*: no significant difference (n=23p=0.12, SMD=−0.30, 95% CI −0.46 to –0.14, p<0.001; reduction in HbA1c, 5 mmol/mol), - *diabetes specialists*: no significant effect (n=30, SMD=−0.18, 95% CI −0.25to –0.10, p<0.001; reduction in HbA1c 3 mmol/mol), - ‘*other’ interventionists*: no significant effect (n=16, SMD=−0.07, 95% CI −0.21 to 0.06, p=0.29; reduction in HbA1c, 1 mmol/mol).   **Heterogeneity**   - *psychology professionals*: high and significant (I2=72.6%, p<0.001) - *diabetes specialists:* moderate for (I2=57.7%, p<0.001) - ‘*other’ interventionists*: moderate (I2=58.2%, p=0.002).   **HbA1c reduction**   - no association between type of primary outcome and change in HbA1c (p=0.33). - no significant difference (meta-regression revealed ) in effect size in HbA1c reduction between studies where HbA1c was a primary outcome (n=33) compared with studies where HbA1c was a secondary outcome (n=37) (p=0.21) 3.   **Most effective stratgies:**   - Self-help materials (58.1%), - CBT (22.4%) - counseling (18.8%), - waiting list control, attention control and usual care were less likely to be the best treatment (all ≤0.6%). |
| 26 | Yang et al.  **Effects of cognitive behavioural therapy–based intervention on improving glycaemic, psychological, and physiological outcomes in adult patients with diabetes mellitus: a meta-analysis of randomized controlled trials** 2020  China | 23 studies  USA (5), Netherlands (4), China (3), Iran (2), UK (2), Australia (1), Belgium (1), Canada (1), Germany (1), Saudi Arabia (1), Sweden (1), Taiwan (1) | RCTs | Diabetes mellitus  Type 1 (7) and type 2 diabetes (12) | 10 (high) | 2,619 | Adherence to treatment  (CBT) | **Total 5**  *Converted to Michie’s taxonomy*  Behavioural experiments (23)  Commitment (3)  Behavioural practice/rehearsal (1)  Motivational interviewing (2)  Instruction on how to perform the behaviour (8)  **As described in the review**:  CBT as a stand-alone intervention (12)  CBT combined with other treatment (11)  acceptance and commitment therapy (3), mindfulness-based cognitive therapy (1).  Motivational interviewing  Education | CBT-based intervention was delivered face-to face in 19 studies, via telephone in two studies, and via internet in two studies.  Most studies conducted a group CBT-based intervention,  individual CBT-based intervention (4). | **Intervention duration**  6 weeks to 1 year | **Overall effect**   - effective with a mean reduction of 0.275% (95% CI: −0.443 to −0.107; p < 0.01) and moderate effect size Hedge’s g 0.466 (95% CI 0.719 to 0.189, p < 0.01). The heterogeneity of these studies was statistically significant (I 2 = 87.007%, p < 0.001)   ***Effect on HbA1c***   - effective   ***Effect on drop-out rate***   - low drop out rate (less than 20%),   **Most effective stratgies**  *HbA1c*   - behavioural experiment, - stress management, - homework assignment - interpersonal strategy. - use of cognitive strategies and mood management   ***Comment:***  There was no difference in the change of fasting plasma glucose, anxiety symptoms, weight, and high-density lipoprotein cholesterol between CBT-based interventions and the control conditions. |
