# Supplementary material for: Effectiveness of behaviour change techniques in lifestyle interventions for non-communicable diseases: an umbrella review
Source: BMC Public Health. 2024 Nov 7;24:3082. doi: 10.1186/s12889-024-20612-8 (PMC11545567; doi:10.1186/s12889-024-20612-8)
Supplement: Supplementary file 3 — Supplementary Material 3 [file 12889_2024_20612_MOESM3_ESM.docx]

Supplementary Table 4: Effective behavioural change techniques among people with Diabetes

| study ID | Intervention | Effective BCT(s) | Clinical outcome measure | | | Behavioural change |
| --- | --- | --- | --- | --- | --- | --- |
|  |  |  | Hx, PE | Lab | Psychological outcomes |  |
| 3 | Treatment adherence | 1,2,5 | . | Fasting Blood Sugar, HbA1c, Total cholesterol, Triglyceride | depression, diabetes distress, psychosocial well-being, self-efficacy, stress, and communication | ↑Health behaviours (diet, physical activity/exercise, medication use, smoking) |
| 15 | Treatment adherence | 1,2,3 | Body weight | HbA1c | psychological distress | . |
| 20 | Treatment adherence | 1,2,5 | . | HbA1c | . | . |
| 25 | Treatment adherence | 2,4 | biomedical (n=5; coronary heart disease risk n=1, BW n=3, BMI n=1). | HbA1c | psychological (n=19; diabetes empowerment n=1, depressive symptoms n=10, diabetes distress n=4, self-efficacy n=2, stress n=2) | self-management behaviours (n=13; physical activity n=6, medication adherence n=5, diet adherence n=2) |
| 26 | Treatment adherence | 2,4,11,13 | Body weight | Fasting Blood Sugar, HbA1c, Total cholesterol, High-Density Lipoproteins | anxiety symptoms | . |
| 9 | Combined | 1,4,6,8 | Body weight | HbA1c | . | . |
| 10 | Combined | 1 | Body weight | HbA1c | Self-efficacy | . |
| 5 | Physical activity | 1,3,4,5,7,15 | BMI | HbA1c | . | ↑Physical activity |
| 14 | Physical activity | no BCT found to have impact | . | . | . | ↑Physical activity |

Abbreviations: BCTs 1- Goals and planning; 2-Feedback and monitoring; 3-Social support; 4-Shaping knowledge; 5-Natural consequences; 6-Compaison of behaviour; 7-Associations; 8-Repetition and substitution; 11-Regulation; 13-Identity; 15-self-belief.
