# Supplementary material for: Effectiveness of behaviour change techniques in lifestyle interventions for non-communicable diseases: an umbrella review
Source: BMC Public Health. 2024 Nov 7;24:3082. doi: 10.1186/s12889-024-20612-8 (PMC11545567; doi:10.1186/s12889-024-20612-8)
Supplement: Supplementary file 4 — Supplementary Material 4 [file 12889_2024_20612_MOESM4_ESM.docx]

Supplementary Table 5: Effective behavioural change techniques among people with Cancer

| study ID | Intervention | Effective BCT(s) | Clinical outcome measure | | | Behavioural change |
| --- | --- | --- | --- | --- | --- | --- |
|  |  |  | Hx, PE | Lab | Psychological outcomes |  |
| 13 | Treatment adherence | 1 | Symptoms (pain, fatigue distress) | . | . | . |
| 6 | Combined | 1,2,3 | . | . | . | ↑Physical activity, ↑diet quality |
| 18 | Combined | 3,4,5 | . | . | some aspects of QOL, such as depression | . |
| 11 | Physical activity | 3,4,7,9,12 | . | . | . | ↑Physical activity |
| 22 | Physical activity | 1,3,8 | . | . | . | ↑Physical activity |

Abbreviations: BCTs 1- Goals and planning; 2-Feedback and monitoring; 3-Social support; 4-Shaping knowledge; 5-Natural consequences; 7-Associations; 8-Repetition and substitution; 9-Comparison of outcomes; 12-Antecedents.
