# Supplementary material for: Effectiveness of behaviour change techniques in lifestyle interventions for non-communicable diseases: an umbrella review
Source: BMC Public Health. 2024 Nov 7;24:3082. doi: 10.1186/s12889-024-20612-8 (PMC11545567; doi:10.1186/s12889-024-20612-8)
Supplement: Supplementary file 5 — Supplementary Material 5 [file 12889_2024_20612_MOESM5_ESM.docx]

Supplementary Table 6: Effective behavioural change techniques among people with Cardiovascular diseases (CVDs) and Respiratory conditions

| study ID | Intervention | Effective BCT(s) | Clinical outcome measure | | | Behavioural change |
| --- | --- | --- | --- | --- | --- | --- |
|  |  |  | Hx, PE | Lab | Psychological outcomes |  |
| Cardiovascular diseases (CVDs) | | | | | | |
| 24 | Treatment adherence | . | BP | . | . | ↑Medical adherence |
| 1 | Physical activity | 2,4 | Systolic and diastolic blood pressure, peak VO2max, 6-minute walk test | Total cholesterol, High-Density Lipoproteins, Low-Density Lipoproteins, | . | . |
| 2 | Physical activity | . | . | . | . | ↑Physical activity |
| Respiratory conditions | | | | | | |
| 4 | Treatment adherence | . | . | . | . | . |
| 23 | Treatment adherence | 2 | ↓ symptoms and unscheduled health care use | . | . | ↑adherence to preventive medication. |
| 8 | Smoking cessation | 1,3,4,5,7 | . | carbon monoxide | . | smoking cessation |

Abbreviations: BCTs 1- Goals and planning; 2-Feedback and monitoring; 3-Social support; 4-Shaping knowledge; 5-Natural consequences; 7-Associations.
