# Supplementary material for: Effectiveness of behaviour change techniques in lifestyle interventions for non-communicable diseases: an umbrella review
Source: BMC Public Health. 2024 Nov 7;24:3082. doi: 10.1186/s12889-024-20612-8 (PMC11545567; doi:10.1186/s12889-024-20612-8)
Supplement: Supplementary file 6 — Supplementary Material 6 [file 12889_2024_20612_MOESM6_ESM.docx]

Supplementary Table 7:: Effective behavioural change techniques among people with Other Noncommunicable diseases (NCDs)

| study ID | Intervention | Effective BCT(s) | Clinical outcome measure | | | Behavioural change |
| --- | --- | --- | --- | --- | --- | --- |
|  |  |  | Hx, PE | Lab | Psychological outcomes |  |
| Musculoskeletal disorders | | | | | | |
| 7 | Physical activity | . | . | . | . | ↑Physical activity adherence |
| 16 | Physical activity | . | . | . | . | ↑Physical activity |
| 17 | Physical activity | . | . | . | . | ↑Physical activity |
| 21 | Physical activity | 1,3,4,6,8 | . | . | . | ↑Exercise adherence |
| Obesity | | | | | | |
| 19 | Combined | 1,2,4,5,8 | Calorie intake, Body weight | . | . | . |
| Chronic Kidney Disease (CKD) | | | | | | |
| 12 | Combined | 3,4 | Blood pressure, peak VO2 | eGFR, sodium or albumin excretion | . | . |

Abbreviations: BCTs 1- Goals and planning; 2-Feedback and monitoring; 3-Social support; 4-Shaping knowledge; 5-Natural consequences; 6-Compaison of behaviour; 8-Repetition and substitution.
