# Supplementary material for: Effectiveness of behaviour change techniques in lifestyle interventions for non-communicable diseases: an umbrella review
Source: BMC Public Health. 2024 Nov 7;24:3082. doi: 10.1186/s12889-024-20612-8 (PMC11545567; doi:10.1186/s12889-024-20612-8)
Supplement: Supplementary file 7 — Supplementary Material 7 [file 12889_2024_20612_MOESM7_ESM.docx]

Supplementary Table 3: Quality Assessment using AMSTAR-2

|  | **AMSTAR questions*** | | | | | | | | | | | | | | | | |
| --- | --- | --- | --- | --- | --- | --- | --- | --- | --- | --- | --- | --- | --- | --- | --- | --- | --- |
| **Title** | **Q1** | **Q2** | **Q3** | **Q4** | **Q5** | **Q6** | **Q7** | **Q8** | **Q9** | **Q10** | **Q11** | **Q12** | **Q13** | **Q14** | **Q15** | **Q16** | **Strength** |
| Behaviour change techniques in home-based cardiac rehabilitation: a systematic review | YES | NO | YES | YES | YES | YES | NO | YES | YES | YES | NO META ANALYSIS | NO META ANALYSIS | YES | YES | YES | YES | High |
| Behaviour change techniques (BCT) inphysical activity interventions formen with prostate cancer: A systematic review | YES | NO | YES | YES | YES | YES | NO | YES | YES | NO | NO META ANALYSIS | NO META ANALYSIS | YES | YES | NO | YES | Moderate |
| Physical activity containing behavioural interventions for adults living with modifiable chronic non-communicable diseases in Africa: a systematic mixed-studies review. | YES | NO | YES | YES | YES | YES | NO | YES | NO | NO | NO META ANALYSIS | NO META ANALYSIS | NO | NO | NO | YES | Modearte |
| Systematic review and meta-analysis of randomised controlled". trials of psychological interventions to improve glycaemic control in patients with type 2 diabetes | YES | NO | YES | YES | YES | YES | NO | YES | YES | YES | YES | YES | YES | YES | YES | YES | High |
| Behaviour change interventions to promote physical activity in rheumatoid arthritis: a systematic review | YES | NO | YES | YES | NO | YES | NO | YES | YES | NO | NO META ANALYSIS | NO META ANALYSIS | YES | YES | NO | NO | Moderate |
| Evaluating the Effect of a Diabetes Health Coach in Individuals with Type 2 Diabetes | NO | YES | YES | YES | YES | YES | NO | YES | YES | NO | YES | YES | YES | YES | YES | YES. | Moderate |
| Identifying active ingredients in complex behavioural interventions for obese adults with obesity-related co-morbidities or additional risk factors for co-morbidities: a systematic review | YES | NO | YES | YES | YES | YES | NO | YES | YES | NO | YES | YES | NO | YES | YES | NO | Moderate |
| Effective Behavior Change Techniques in Asthma Self-care Interventions: Systematic Review and Meta-Regression | YES | NO | YES | YES | NO | YES | YES | YES | YES | YES | YES | YES | YES | YES | YES | YES | HIgh |
| Systematic review and meta-analysis of maintenance of physical activity behaviour change in cancer survivors | YES | YES | PARTIALLY | YES | NO | YES | NO | YES | YES | NO | YES | YES | YES | YES | NO | YES | Moderate |
| Behaviour change techniques associated with adherence to prescribed exercise in patients with persistent musculoskeletal pain: Systematic review | YES | NO | YES | YES | YES | YES |  | YES | YES | NO | NO META ANALYSIS | NO META ANALYSIS | YES | YES | NO | NO | Moderate |
| Lifestyle behaviour change for preventing the progression of chronic kidney disease: a systematic review | YES | YES | YES | YES | YES | YES | YES | YES | YES | NO | NO META ANALYSIS | NO META ANALYSIS | YES | YES | YES | YES | high |
| ﻿Goal-setting in diabetes self-management: A systematic review and meta-analysis examining content and effectiveness of goal-setting interventions | PARTIALLY | NO | YES | YES | NO | YES | NO | YES | YES | NO | YES | YES | YES | YES | YES | YES | Moderate |
| Effective behaviour change techniques in smoking cessation interventions for people with chronic obstructive pulmonary disease: A meta-analysis | PARTIALLY | NO | YES | YES | NO | YES | NO | YES | YES | NO | YES | YES | YES | YES | YES | NO | Moderate |
| How Behavior Change Strategies are Used to Design Digital Interventions to Improve Medication Adherence and Blood Pressure Among Patients With Hypertension: Systematic Review. | Yes | YES | yes | Yes | YES | YES | yes | YES | YES | NO | NO META ANALYSIS | NO META ANALYSIS | YES | yes | yes | YES | high |
| ﻿Behavior change theory, content and delivery of interventions to enhance adherence in chronic respiratory disease: A systematic review | NO | NO | PARTIALLY | YES | YES | YES | NO | YES | YES | NO | NO META ANALYSIS | NO META ANALYSIS | NO | NO | NO | YES | Low |
| ﻿Use of Behavioral Change Techniques in Web-Based Self-Management Programs for Type 2 Diabetes Patients: Systematic Review | YES | NO | YES | YES | YES | YES | NO | YES | YES | NO | NO META ANALYSIS | NO META ANALYSIS | NO | NO | NO | NO | Low |
| "Behavior Change Techniques in Physical Activity eHealth Interventions for People With Cardiovascular Disease: Systematic Review | PARTIALLY | NO | YES | YES | YES | YES | NO | YES | YES | NO | YES | YES | YES | YES | YES | YES | Moderate |
| Effects of Cognitive Behavioral Therapy–Based Intervention on Improving Glycaemic, Psychological, and Physiological Outcomes in Adult Patients With Diabetes Mellitus: A Meta-Analysis of Randomized Controlled Trials | YES | NO | YES | YES | YES | YES | NO | YES | YES | NO | NO META ANALYSIS | NO META ANALYSIS | YES | YES | YES | YES | Moderate |
| Behaviour change techniques applied in interventions to enhance physical activity adherence in patients with chronic musculoskeletal conditions: A systematic review and meta-analysis | YES | YES | YES | YES | NO | YES | NO | YES | YES |  | YES | YES | YES | YES | YES | YES | Moderate |
| A systematic review and meta-analysis of social cognitive theory-based physical activity and/or nutrition behavior change interventions for cancer survivors" | YES | YES | YES | Yes | NO | YES | NO | YES | YES |  | YES | YES | YES | YES | YES | YES | Moderate |
| Changing Physical Activity Behavior in Type 2Diabetes A systematic review and meta-analysis of behavioral interventions" | YES | YES | YES | Yes | YES | Yes | NO | NO | YES |  | NO META ANALYSIS | NO META ANALYSIS | YES | yes | yes | YES | Moderate |
| A systematic review of the effectiveness of problem-solving approaches towards symptom management in cancer care | YES | NO | YES | YES | Can't Answer | YES | NO | YES | NO |  | NO META ANALYSIS | NO META ANALYSIS | No | NO | NO | Yes | Low |
| Behaviour change interventions targeting physical activity in adults with fibromyalgia: a systematic review | PARTIALLY | YES | YES | YES | NO | YES | NO | YES | YES |  | NO META ANALYSIS | NO META ANALYSIS | YES | YES | YES | YES | Moderate |
| Counseling interventions delivered in women with breast cancer to improve health-related quality of life: a systematic review | YES | NO | YES | YES | YES | NO | NO | YES | NO |  | YES | PARTIALLY | NO | PARTIALLY | PARTIALLY | YES | Moderate |
| Psychological interventions to improve glycemic control in adults with type 2 diabetes: a systematic review and meta- analysis | YES | YES | YES | YES | YES | YES | NO | YES | YES |  | YES | YES | YES | YES | YES | YES | High |
| ﻿Behaviour change techniques targeting both diet and physical activity in type 2 diabetes: A systematic review and meta-analysis | YES | NO | YES | YES | YES | YES | NO | YES | YES |  | YES | YES | YES | YES | NO | YES | Moderate |

*List of questions in AMSTAR-2 tool; Q1 Did the research questions and inclusion criteria for the review include the components of PICO?; Q2 Did the report of the review contain an explicit statement that the review methods were established prior to the conduct of the review and did the report justify any significant deviations from the protocol?; Q3 Did the review authors explain their selection of the study designs for inclusion in the review?; Q4 Did the review authors perform study selection in duplicate?; Q5 Did the review authors perform data extraction in duplicate?; Q6 Did the review authors use a comprehensive literature search strategy?; Q7 Did the review authors provide a list of excluded studies and justify the exclusions?; Q8 Did the review authors describe the included studies in adequate detail?; Q9 Did the review authors use a satisfactory technique for assessing the risk of bias (RoB) in individual studies that were included in the review; Q10 Did the review authors report on the sources of funding for the studies included in the review?; Q11 If meta-analysis was performed did the review authors use appropriate methods for statistical combination of results? RCTs/NRSI; Q12 If meta-analysis was performed, did the review authors assess the potential impact of RoB in individual studies on the results of the meta-analysis or other evidence synthesis?: Q13 Did the review authors account for RoB in individual studies when interpreting/ discussing the results of the review?; Q14 Did the review authors provide a satisfactory explanation for, and discussion of, any heterogeneity observed in the results of the review?; Q15 If they performed quantitative synthesis did the review authors carry out an adequate investigation of publication bias (small study bias) and discuss its likely impact on the results of the review?: Q16 Did the review authors report any potential sources of conflict of interest, including any funding they received for conducting the review?
